# A critical evaluation of QIDS-SR-16 using data from a trial of psilocybin therapy versus escitalopram treatment for depression

**DOI:** 10.1177/02698811231167848

**Published:** 2023-04-25

**Authors:** Brandon Weiss, David Erritzoe, Bruna Giribaldi, David J Nutt, Robin L Carhart-Harris

**Affiliations:** 1 Centre for Psychedelic Research, Division of Academic Psychiatry, Imperial College London, London, UK; 2 Psychedelics Division, Neuroscape, Department of Neurology, University of California, San Francisco, CA, USA

**Keywords:** Clinical trial, depression measurement, escitalopram, psilocybin therapy, QIDS

## Abstract

**Background:**

In a recent clinical trial examining the comparative efficacy of psilocybin therapy (PT) versus escitalopram treatment (ET) for major depressive disorder, 14 of 16 major efficacy outcome measures yielded results that favored PT, but the Quick Inventory of Depressive Symptomatology, Self-Report, 16 items (QIDS-SR_16_) did not.

**Aims:**

The present study aims to (1) rationally and psychometrically account for discrepant results between outcome measures and (2) to overcome psychometric problems particular to individual measures by re-examining between-condition differences in depressive response using all outcome measures at item-, facet-, and factor-levels of analysis.

**Method:**

Four depression measures were compared on the basis of their validity for examining differences in depressive response between PT and ET conditions.

**Results/Outcomes:**

Possible reasons for discrepant findings on the QIDS-SR_16_ include its higher variance, imprecision due to compound items and whole-scale and unidimensional sum-scoring, vagueness in the phrasing of scoring options for items, and its lack of focus on a core depression factor. Reanalyzing the trial data at item-, facet-, and factor-levels yielded results suggestive of PT’s superior efficacy in reducing depressed mood, anhedonia, and a core depression factor, along with specific symptoms such as sexual dysfunction.

**Conclusion/Interpretation:**

Our results raise concerns about the adequacy of the QIDS-SR_16_ for measuring depression, as well as the practice of relying on individual scales that tend not to capture the multidimensional structure or core of depression. Using an alternative approach that captures depression more granularly and comprehensively yielded specific insight into areas where PT therapy may be particularly useful to patients and clinicians.

## Introduction

**Figure 1.**
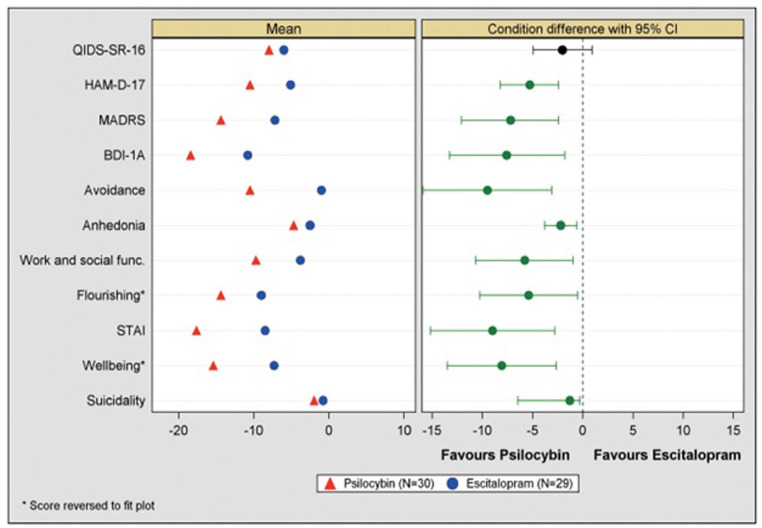
All (mean change) efficacy outcomes compared between conditions at week 6 (primary endpoint). ET in blue, psilocybin in red. Green CIs indicate no crossing of zero (i.e., >95% confidence in difference), black CIs indicate crossing of zero and hence no between-condition statistical difference. Left panel is mean, right panel is mean difference and 95% CI. Source: Directly reproduced from Carhart-Harris et al. (2021), that is, Figure S6 Supplemental Appendix. CI: confidence interval; ET: escitalopram treatment.

In a recent clinical trial examining the comparative mechanisms and efficacy of psilocybin treatment (PT) versus escitalopram treatment (ET) for major depressive disorder (MDD) (Carhart-Harris et al., 2021; Daws and Carhart-Harris, 2022), 14 of 16 major efficacy outcome measures yielded results that favored the PT arm with greater than 95% confidence, but two did not (source data shown in Table 2 of the main clinical paper, plus Supplemental Figure S4—which is reproduced here as Figure 1). Both negative results came from the Quick Inventory of Depressive Symptomatology, Self-Report, 16 items (QIDS-SR_16_) (Rush et al., 2003). Since every efficacy outcome measure in this trial favored PT except for QIDS-SR_16_ outcomes, we felt motivated to ask whether the negative results on QIDS-SR_16_ data were possibly related to this scale’s inability to detect a “true” between-condition difference. As mean change on the QIDS-SR_16_ was this study’s pre-registered primary depression-related outcome measure, the null finding dominated the framing of the published study report, with readers editorially instructed to draw no conclusions on the study’s data in terms of PT’s efficacy relative to ET.

We believe that probing the origin of the discrepancy between the “miss” on the primary outcome and the “hits” (i.e., efficacy results significantly favoring PT) on the remaining efficacy outcome measures is a legitimate matter of scientific investigation that could have specific and general implications; *specific*, in relation to how to best interpret the findings of the Carhart-Harris et al. Carhart-Harris et al. (2021) trial, and *general*, in relation to use of the QIDS-SR_16_ in other research studies.

Valid assessment of treatment-related symptom change is critical to the validity of information yielded by clinical trial design. Given the considerable societal burden and harms related to depression (Funk, 2016), striving to improve measurement validity is important for scientific advancement in depression research and treatment, as is the discovery of better treatments. One area where several current depression rating scales have been argued to be weak is in their use of sum-scoring all items, as if they all relate to one single internally consistent dimension, that is, a “depression” dimension (Fried et al., 2022). As we shall see in the next sections, this approach is particularly problematic if a scale’s array of items lacks sufficiently high internal consistency and specificity to the core of depression, where “core” is defined by being comprised by depression’s most causally central symptoms and being most related to psychosocial impairment. As a brief note, we do not regard the idea of a “core” factor of depression as mutually exclusive with idiographic approaches to psychopathology that recognize the unique causal interplay of symptoms that characterize depression for different individuals (Fisher et al., 2017).

## The IDS and the origin of the QIDS-SR_16_

The present analysis is focused on the validity of the QIDS-SR_16_ (Rush et al., 2003). The QIDS-SR_16_ was first presented in 2003 as a shorter version of its predecessor, the Inventory of Depressive Symptoms or IDS-SR (Rush et al., 1986). We believe that the original motivation for and methods of validating the IDS, are worth considering as we critically evaluate the QIDS-SR_16_ in what follows.

The IDS was first published in 1986 and was inspired by a desire to be inclusive of atypical presentations of depression including those characterized by hypersomnia and weight gain (Rush et al., 2000). In its original validation paper, the IDS furthermore introduced a four-factor model of depression, a structure that lost emphasis over time. The use of unifactor scoring may have been accelerated with the introduction of the QIDS-SR_16_, a scale that was devised to be simple and brief. The QIDS-SR_16_ is intentionally faithful to the nine Diagnostic and Statistical Manual of Mental Disorders (American Psychiatric Association, 2010; American Psychiatric Association, 2013) criteria for MDD. Indeed, QIDS-SR_16_ was selected as the primary outcome measure in our original trial based on its use in the large-scale prospective depression study, Sequenced Treatment Alternatives to Relieve Depression (STAR*D) (Trivedi et al., 2006), and its convenience as a short scale that can be administered frequently without heavy patient burden.

However, recent commentators have argued and provided evidence for the view that the DSM definition of depression may insufficiently capture a “core,” causally central depression factor (Fried et al., 2016a) most strongly characterized by psychosocial impairment (Fried and Nesse, 2014). In seeking to capture atypical depressive subtypes, the IDS, subsequent QIDS-SR_16_, and DSM-5 may miss an opportunity to narrow in on more “core” dimensions or factors of depression comprising symptoms that are the most mechanistically relevant to identify and intervene on.

## Assessing the validity of the QIDS-SR_16_

Prior assessments of the validity of the QIDS-SR_16_ have shown it to exhibit good validity in some but not all domains (Reilly et al., 2015). For example, of the more than 40 studies that have evaluated the psychometric properties of QIDS-SR_16_ (Reilly et al., 2015), just 3 have examined its test-retest reliability. This is somewhat surprising given that there are certain attributes of the QIDS-SR_16_ that place it at high risk for poor test-retest performance.

For example, the QIDS-SR_16_ contains a high number of *compound* items, where a single item contains two or more individual depression symptoms. According to Fried (2017), 90% of the QIDS-SR_16_’s items can be considered compound, compared with 45% (Hamilton Rating Scale for Depression-17 (HRS); Hamilton, 1960), 42% (Montgomery and Asberg Depression Rating Scale (MADRS); Montgomery and Åsberg, 1979), and 24% (Beck Depression Inventory-IA (BDI_IA_); Beck et al., 1996) in other widely used measures.

There are two forms of compound items within the QIDS-SR_16_. The first involves items that contain within it two distinct, but related symptoms (e.g., QIDS-SR_16_ item 10 encompassing concentration and decision-making difficulties). Otherwise known as “double-barreled” (Johns, 2010), such content permits two participants to interpret a single item in substantively different ways through attending to different individual symptoms within it. This variability in interpretation can amount to increased variability between participants in the construct being measured and variance in the sum-scores. Although the presence of multiple individual symptoms within an item would not be particularly concerning in cases where individual symptoms are well correlated, individual depression symptoms can be quite divergent from each other (Fried et al., 2016a). In addition, given that individual symptoms differ considerably in their causal centrality among depressive symptoms (Fried et al., 2016a) and their association to impairment (Fried and Nesse, 2014), inclusion of two individual symptoms that differ in these regards can substantially impact the clinical relevance of the scale’s overall sum-score.

The second form of compound item within the QIDS-SR_16_ magnifies these problems. The QIDS-SR_16_ was designed to match the DSM criteria for MDD. Whereas six of the nine criteria are indexed by single items, the QIDS-SR_16_ is unique among other widely used depression measures in directing raters to select the highest-scored item among multiple items to index three ancillary criteria: sleep problems (highest among four), weight/appetite problems (highest among four), and psychomotor problems (highest among two) (see Table 1 for item descriptions). This compound nature of the QIDS-SR_16_ may have resulted from its abbreviation from its predecessor, the IDS-SR, which contained 30 items and generally expressed all item scores in the subscale scores rather than selecting only the highest-scored items.

**Table 1.** Description of compound criterion items.

Sleep	QIDS1	*Falling asleep*
	QIDS2	*Sleep during night*
	QIDS3	*Wake up too early*
	QIDS4	*Sleep too much*
Weight/appetite	QIDS6	*Decreased appetite*
	QIDS7	*Increased appetite*
	QIDS8	*Decreased weight*
	QIDS9	*Increased weight*
Psychomotor	QIDS15	*Feeling slowed down*
	QIDS16	*Feeling restless*

QIDS: Quick Inventory of Depressive Symptomatology.

This compound scoring practice would be psychometrically questionable if the items that make up each domain showed poor internal consistency and differed widely in their clinical relevance, and there is some suggestion that this may be the case. Sleep problems, weight/appetite problems, psychomotor problems each encompass opposite features (insomnia and hypersomnia; weight/appetite gain and loss; psychomotor retardation and agitation), and a 2016 meta-analysis observed that sleep and appetite items showed unacceptable item-total correlations (*r* > 0.30) in five and three studies, respectively. Both forms of compound items, but particularly the latter form (heretofore *compound criteria*), may impact test-retest reliability in the context of prospective measurement. This could be especially true for cases in which the item that participants score highest on differs between the two timepoints, and the two items are not well-correlated.

Previous research from the large STAR*D dataset (Trivedi et al., 2006) is suggestive of weak to moderate intercorrelations between QIDS-SR_16_ hyposomnia items (0.16 < *r* < 0.47), a moderate intercorrelation between QIDS-SR_16_ appetite and weight items (*r* = 0.33; though a correlation between decreased and increased weight/appetite scores could not be computed based on the data), and a weak intercorrelation between psychomotor criterion items (*r* = 0.22; from Fried et al., 2016b, supplementary).

## Test-retest validity of the QIDS-SR_16_

Of the three studies that have examined the QIDS-SR_16_’s test-retest reliability over an approximately 2-week period, estimates range from 0.49 to 0.77 (Hong et al., 2013; Ma et al., 2015; Zhang et al., 2020). These estimates are considered to show suboptimal measurement error by most (Cicchetti, 1994), but not all guidelines (Fleiss, 2011). A review of the literature suggests that these estimates may be inferior to test-retest estimates respecting other depression measures including the MADRS—for example, intra-class correlation (ICC) > 0.93_3–14days_ (Ahmadpanah et al., 2016); ICC_1wk_ = 0.88 (Yee et al., 2015); HRS—for example, meta-analyzed ICC = 0.94, *r* = 0.87 (Trajković et al., 2011); BDI_IA_ (Beck et al., 1961)—for example, ICC_2wks_ = 0.89 (Visser et al., 2006), and BDI_II_—for example, *r*_1–12days_ = 0.83 (Sprinkle et al., 2002).

The QIDS-SR_16_ ICC scores are lower than all the above; however, the test-retest time periods for these estimates varied widely, and reliability is known to decline over larger periods (Trajković et al., 2011). A formal meta-analysis would be required to make valid comparisons. Nevertheless, given the foregoing psychometric concerns, the low number of studies examining the QIDS-SR-16’s test-retest reliability, and the presence of suboptimal reliability across known estimates, it is believed that the QIDS-SR_16_ deserves greater psychometric scrutiny on the test-retest domain. Poor test-retest reliability on the QIDS-SR_16_ would imply that this scale has a poor signal-to-noise-ratio, affecting the scale’s ability to measure MDD-related symptom severity sensitively and reliably.

Although antidepressant response is typically measured using scale sum-scores as in QIDS-SR_16_ scoring, a substantial body of literature cogently indicates that depression can be more validly measured in a multidimensional fashion that respects individual symptoms and/or depression facets as clinically relevant outcomes of interest (Fried et al., 2022). Indeed, as early as 1960, Hamilton referred to the sum-score as the “total *crude* score,”and favored analyzing depression at a narrower subscale level of analysis (Hamilton, 1960). Recent findings show that depression is heterogeneous and multidimensional both within individual scales (Bagby et al., 2004; Shafer, 2006) and across symptoms (Ballard et al., 2018; Fried et al., 2016a; Gullion and Rush, 1998), individual symptoms differ in their biological correlates (Fried and Nesse, 2015; Jang et al., 2004), individual symptoms differ in their response to the same treatment (Hieronymus et al., 2016a, 2016b; Lamers et al., 2013; Thase, 2002), and not all symptoms are equivalent with respect to their causal centrality to depression (Fried et al., 2016a) or their associated level of impairment to functioning (Fried and Nesse, 2014). As a note, even the IDS-SR demonstrated a four-factor structure (Rush et al., 1986), which was arguably neglected when moving to the shorter QIDS-SR_16_.

A consequence of using sum-scores despite multidimensionality in the underlying construct is that relative improvement in one symptom or facet of depression may be masked by poor improvement in other less clinically relevant domains. The question before us is whether this could be the case with the QIDS-SR_16_, if, for example, its items and scoring deviate from core components of depression.

Sum-scores also vary considerably from each other in terms of symptom content being measured (Fried, 2017), and it is not clear that scales that match DSM criteria, such as the QIDS-SR_16_, are more clinically relevant than scales that do not. DSM taxonomies have been critiqued and seem unlikely to capture core symptomatology (Beam et al., 2021). Indeed, the QIDS-SR_16_ was devised to be faithful to the standard diagnostic definition of MDD in measuring all nine DSM-5 criteria (Rush et al., 2000), whereas the BDI_IA_ only contains six of nine criteria (Moran and Lambert, 1983), excluding symptoms related to increased appetite, hypersomnia, and psychomotor activity and agitation. Previous research has shown that DSM symptoms are not more causally central to depression than non-DSM symptoms (Fried et al., 2016a), and DSM criteria excluded from the BDI_IA_ are among the least relevant to psychosocial impairment (Fried and Nesse, 2014). In addition to this, many scales, including the HRS and BDI_IA_, have been criticized for poor psychometric properties, including poor inter-rater reliability, content validity, and item functioning (Bagby et al., 2004; Gullion and Rush, 1998).

A possible solution to the problems attending researchers’ reliance on sum-scores is to focus on more granular levels of analysis, namely on individual symptoms or correlated clusters of symptoms, that is, “depression facets.” Such a move is in line with network and process-based biopsychosocial models of psychopathology, which highlight complex interactions between causes and effects of symptoms of mental illness (Borsboom and Cramer, 2013; Hayes and Hofmann, 2017; Kočárová et al., 2021; Wade and Halligan, 2017) and challenge the precision and validity of current diagnostic categories that specify latent causes for underlying symptoms (Insel et al., 2010).

## A trial of PT versus ET for depression

Given these concerns about the QIDS-SR_16_ and scale sum-scores more broadly (Fried et al., 2022), the present study examines the psychometric properties of the QIDS-SR_16_ using the Carhart-Harris et al. (2021) clinical trial data of PT versus ET as a case study. It performs two exploratory approaches to evaluate the efficacy of PT versus ET in the trial.

In the first set of analyses, we examine the psychometric functioning of the QIDS-SR_16_ scale relative to other depression scales. In the second set of analyses, we examine between-condition response in newly computed outcomes. The latter analyses are in line with calls for more granular measurement of depression that respects its heterogeneous structure and affords identification of differential symptom response to treatment. Two approaches were undertaken. First, Ballard et al.’s (2018) factor structure of depression is used to examine granular facets of depression from our data. Relative efficacy of PT versus ET is subsequently tested across these outcomes to understand which depression facets are most sensitive to differential response. Ballard et al.’s factor structure was selected due to its methodological rigor and unique selection of scales that almost perfectly corresponded with the present study. Performing our own exploratory factor analysis (EFA) was considered, but rejected given the inadequacy of our sample size.

Second, in line with calls to measure depression using individual symptoms with highest causal centrality (Fried et al., 2016a), a single depression factor is derived (using EFA) comprised of those items that best reflect the *core* of the four depression scales that were used in the Carhart-Harris et al. trial. Relative efficacy of PT versus ET was subsequently tested using this *core* depression factor.

Finally, it bears noting that the present study is intended to be a good-faith effort to understand the source of discrepancy among the depression scales used in the Carhart-Harris et al. (2021) trial, and additionally to probe how individual symptoms and facets of depression may differentially respond to PT versus ET and vice versa. Post hoc analyses undertaken here are known to attend type I error, and thus are cautiously undertaken in exploratory fashion.

## Method

Information regarding trial ethics, patient characteristics, and inclusion/exclusion criteria can be found in the original Carhart-Harris et al. (2021) article (ClinicalTrials.gov number, NCT03429075). Briefly, 59 patients with diagnoses of MDD were randomized to either the PT arm (*N* = 30) or the ET arm (*N* = 29). Written informed consent was obtained from all patients. At visit 1 (baseline), patients provided written informed consent, and completed self-report questionnaires and clinician-rated interviews. At visit 2 (one day after visit 1), the patients in the PT group received 25 mg of COMPASS Pathways’ investigational, proprietary, synthetic, psilocybin formulation, i.e., COMP360, and those in the ET group received 1 mg of psilocybin. All investigators and medication-administering staff were unaware of trial-group assignment. At the end of visit 2, patients received a bottle of capsules and were instructed to take one capsule each morning until their next scheduled day of psilocybin dosing. The capsules contained either microcrystalline cellulose (placebo), which were given to the patients who received the 25 mg dose of psilocybin or 10 mg of escitalopram, which were given to patients who received the 1 mg dose of psilocybin. Three weeks after the first dosing session (visit 2), patients received their second dose of 25 mg psilocybin or 1 mg psilocybin, and patients were instructed to take two capsules each morning (either placebo in PT group or an increased dose of 20 mg of escitalopram in the ET group) for the next 3 weeks. Following 3 weeks, the patients returned to complete self-report questionnaires and clinician-rated interviews.

The authors assert that all procedures contributing to this work comply with the ethical standards of the relevant national and institutional committees on human experimentation and with the Helsinki Declaration of 1975, as revised in 2008. All procedures involving human subjects/patients were approved by the Brent Research Ethics, Committee, the UK Medicines and Healthcare, Products Regulatory Agency, the Health Research Authority, the Imperial College London, Joint Research Compliance and General Data, Protection Regulation Offices, and the risk assessment and trial management review board at the trial site (the National Institute for Health Research Imperial Clinical Research Facility). COMPASS Pathways provided psilocybin (as COMP360). The Pharmacy Manufacturing Unit at Guy’s and St Thomas’s Hospital provided escitalopram and placebo capsules.

### Measures

#### Primary clinical outcome

The 16-item QIDS-SR_16_ (Rush et al., 2000) was created as a version of the IDS-SR with four main goals in mind: (1) to reduce patient burden with a shorter measure, (2) to match more closely the DSM criteria of MDD, (3) to reflect atypical presentations of depression involving hypersomnia and weight gain, and (4) to reduce the weighting of cognitive symptoms as instantiated in the BDI (Rush et al., 1986). The QIDS-SR_16_ was used to measure weekly changes in depression following baseline until 6 weeks end point. Scores measured at baseline, 5 weeks, and 6 weeks post treatment inception will be used in this study. Six weeks was the primary study end point. The traditional QIDS-SR_16_ sum-score contains the sum of nine items that closely match the DSM-5 criteria for MDD. Of the 16 items, 4 are related to sleep problems, 4 are related to weight/appetite problems, and 2 are related to psychomotor problems. From each of these clusters of items, the highest-scored item is selected and summed with the other six individual items to compute the sum-score. Internal consistency was α = 0.75 for baseline and α = 0.89 at 5 and 6 weeks. All QIDS-SR_16_ items are contained in Supplemental Table S8 for reference.

In addition, two new composites were computed to evaluate QIDS-SR_16_ psychometric functioning without its compound criteria. *QIDS-SR_16_ all item 1* averages all individual items except for *QIDS Sleeping too much*, *QIDS Increased appetite*, and *QIDS Increased weight. QIDS-SR_16_ all item 2* averages all individual items except for *QIDS Sleeping too much*, *QIDS Decreased appetite*, and *QIDS Decreased weight*. These two composites were computed because averaging across “increased” and “decreased” items within the sleep items and weight/appetite items would have caused psychometric problems without reverse-scoring.

#### Secondary clinical outcomes

An additional three depression scales and one anhedonia scale were used together with the QIDS-SR_16_ to compute depression facet scores based on Ballard et al.’s (2018) factor structure. The four depression scales were used to derive a single factor score (see below). These measures included the MADRS (Montgomery and Åsberg, 1979), a 10-item clinician-administered depression scale (α_T1(Baseline)_ = 0.65, α_6wks_ = 0.91), the HRS (Hamilton, 1960), a 17-item clinician-administered depression scale (α_T1_ = 0.15, α_6wks_ = 0.81), the BDI_IA_ (Beck et al., 1961), a 21-item self-report scale of depression (α_T1_ = 0.75, α_6wks_ = 0.94), and the Snaith-Hamilton Pleasure Rating Scale (Snaith et al., 1995), a 14-item self-report measure of anhedonia (α_T1_ = 0.85, α_6wks_ = 0.96).

#### Narrow depression facets

A factor analysis was computed through allocating each of the 78 items from the five scales administered in the present trial to one of Ballard et al.’s (2018) EFA-derived factors/subscales. This computation was made possible by virtue of substantial convergence between depression scales administered in this trial (HRS, MADRS, SHAPS, BDI_IA_, QIDS-SR_16_) and those administered by Ballard et al. (2018) (HRS, MADRS, SHAPS, BDI_II_). In the first step, we placed items from different measures on the same 0–1 scale by dividing each item score by the “points-possible” on the item (i.e., a score of 2 on a 1–4 scale was transformed to 0.50). In the second step, we allocated our items to Ballard’s factors through (a) reference to Ballard’s item-factor structure (for convergent items) and (b) rational analysis of QIDS-SR_16_ and BDI_IA_ items’ relevance to Ballard et al.’s factors (for new items). Baseline items were excluded for which no more than five patients endorsed an item above the lowest response choice. Additionally, items were excluded for which no factor seemed directly relevant. In the third step, during tests of internal consistency, items were excluded that exhibited *r*.drop < 0.20 (i.e., items whose correlation with the factor total score [without the item] was lower than 0.20). Of note, Ballard et al.’s Tension factor was excluded due to containing just two items following the aforementioned exclusion rules, and inadequately reflecting the original factor Ballard et al. had derived. Resulting narrow depression facet scores included *Amotivation* (α_T1_ = 0.74, 0.94), *Reduced Appetite* (α_T1_ = 0.83, α_6wks_ = 0.74), *Impaired Sleep* (α_T1_ = 0.77, α_6wks_ = 0.82), *Suicidal Thoughts* (α_T1_ = 0.86, α_6wks_ = 0.92), *Negative Cognition* (α_T1_ = 0.66, α_6wks_ = 0.90), *Depressed Mood* (α_T1_ = 0.76, α_6wks_ = 0.94), and *Anhedonia* (α_T1_ = 0.83, α_6wks_ = 0.95). Supplemental Table S1 describes our item-factor structure as well as reasons for item exclusion. Supplemental Table S2 provides correlations between granular domain scores at baseline. Supplemental Materials I describes the construct validity of these facets.

#### Depression factor score

Exploratory factor analyses were conducted to derive a single latent factor reflecting shared variance across the four main depression scales (QIDS-SR_16_, BDI_IA_, HRS, MADRS). The SHAPS was not included here because it is not regarded as a holistic index of depression. Specifically, items and item composites were forced to load on one factor comprising all items; accordingly, highest loading items/composites were those that explained the largest amount of variance in the overall factor. Although sample size was low (*N* = 57), conditions were considered acceptable (i.e., high λ, single factor, high number of variables; de Winter et al., 2009).^1^

In the first step, we placed items from different measures on the same 0–1 scale by dividing each item score by the “points-possible” on the item (i.e., a score of 2 on a 1–4 scale was transformed to 0.50).

In the second step, we reduced the number of variables in the model to support a positive-definite correlation matrix under low sample size conditions. To do so, items from each depression scale and each Ballard et al. factor were averaged together to create item composites. Supplemental Table S3 contains these composite structures.

In the third step, two HRS items (Weight Loss, Insight) were excluded due to low variability (i.e., less than six patients endorsed these items above the lowest response choice). Factor analyses were subsequently conducted to extract one factor using the Ordinary Least Squares factoring method (see Supplemental Table S4 for factor loadings). The factor accounted for 15% of the variance in the items/composites. Factor loadings were suggestive that the depression factor primarily captures facets of depression including depressed mood, negative self-appraisal, and amotivation. Factor scores were computed for the two timepoints, separately, by creating a mean-score of items/composites loading above 0.40 on the factor. Depression factor scores are therefore on a 0–1 scale. Internal consistency was α = 0.84 for baseline and α = 0.95 at 6 weeks. Supplemental Table S2 provides correlations between this single factor score and the granular factor scores described above.

#### Expectancy

Treatment response expectancies were measured the day before the first dosing day with two questions asking patients about the degree of improvement they would predict after receiving PT and ET separately: For ET: “At the end of the trial after receiving escitalopram every day for 6 weeks, how much improvement in your mental health do you think will occur?” For PT:

Please rate the following with regards to the prospect of receiving two full strong doses of psilocybin, 3 weeks apart. At the end of the trial, 3 weeks after your second PT dosing session, how much improvement in your mental health do you think will occur?

Each of these variables was measured on a 100-point scale. To examine the relative expectancy of improvement by PT versus ET, a new variable was computed (Relative expectancy) involving the subtraction of ET expectancy from PT expectancy. This variable will be used as an index of relative expectancy and a partial proxy for placebo effect predisposition. Expectancy data was available for 55 patients.

### Analytic plan

Two sets of analyses were planned. The first set of analyses examined the psychometric functioning of the QIDS-SR_16_ scale. Linear mixed effects (LME) models were conducted using R software (package “lme4”), in which all items from four depression scales were separately regressed onto the interaction of *Time* and *Condition*, *with a random effect of intercept specified*. The interaction coefficient (*Time* × *Condition*) was used as an index of between-condition differences in unstandardized item score change between baseline and subsequent timepoints.

First, to understand which symptoms are most differentially responsive to the two treatments, items were identified across scales that exhibited strongest differential response. To examine its sensitivity to between-condition change, the QIDS-SR_16_ was then evaluated on the degree these most differentially responsive symptoms were represented.

Second, estimates of between-condition response in item-level change were then used to compare QIDS-SR_16_ items to similar items from other scales that would be expected to show similar results. Each item was placed on the same response scale by dividing each patients’ score by the “points-possible” on the item (i.e., number of response choices for a given item). In cases of discrepancy, QIDS-SR_16_ items were rationally analyzed to observe any differences in the content of the items that could explain differential results relative to other scale items. The BDI_IA_ was considered the most appropriate for comparison for two reasons, namely its comparable self-report format and its insulation from clinician expectancies favorable to PT which may have played a role in clinician-rated measurement. However, unlike the MADRS and HRS, the BDI_IA_ asked patients to report on their symptoms within a longer preceding timeframe than the QIDS-SR_16_, namely 2 weeks versus 1 week. Therefore, BDI_IA_ items were compared to QIDS-SR_16_ items measured at 5 weeks and 6 weeks following the first dose session, whereas MADRS and HRS items were compared to QIDS-SR_16_ items measured at 6 weeks.

Third, three properties of each QIDS-SR_16_ compound criterion were examined including (a) the frequency with which patients rated a different item with the highest score at baseline versus six weeks (inconsistency), (b) the intercorrelations between the item scores at baseline that make up each criterion, and (c) the intercorrelations between the item change scores across timepoints among the items that make up each criterion. Compound criteria were interpreted to exhibit potential measurement error where inconsistency was high and intercorrelations of scores were low.

Fourth, LME models were separately conducted to observe the standard error of the *Time* × *Condition* interaction term coefficient for the four depression scale scores. To place all scale scores on the same response scale, item scores were divided by the number of response choices and item scores that comprise each scale score were averaged (producing scale mean-scores). The standard deviation of baseline scale mean-scores and the standard deviation of changes in scale mean-scores over time were additionally examined to explore possible sources of error.

The second set of analyses examined between-condition response in newly computed outcomes (i.e., seven narrow depression facets, EFA-derived depression factor). LME models were conducted in which each factor score was separately regressed onto the interaction of *Time* and *Condition*. The interaction coefficient (*Time* × *Condition*) was used as an index of differential treatment response at 6 weeks. In addition, to further control for the influence of expectancy, for models that contained a significant interaction term, supplementary models were conducted in which each outcome was separately regressed onto a *Time* × *Condition×* *Relative Expectancy* interaction (Supplemental Materials II). Across sets of analyses, standardized (*b*) and unstandardized (*B*) coefficients are provided to describe LME interaction coefficients. The standardized coefficients reflect the difference between conditions in normalized scores of the outcome; the unstandardized coefficients reflect the difference between conditions in unaltered scores of the outcome (i.e., scores based on the response option scale). The statistical significance threshold was set at *p* < 0.05, two-tailed.

## Results

### Examining the psychometric properties of the QIDS-SR_16_

#### Examining most differentially responsive symptoms

**Figure 2.**
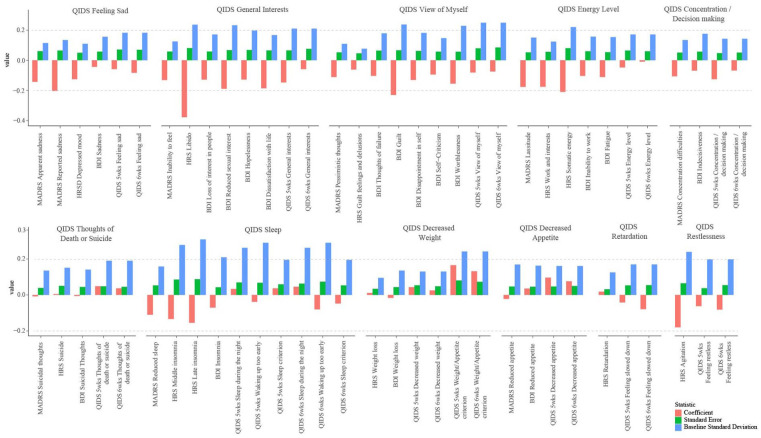
Item-level comparison.

Items were identified from the four depression scales that exhibited strongest differential response to the present treatments, and we examined the degree to which these symptoms are represented within the QIDS-SR_16_ scale. Figure 2 illustrates estimates of between-condition differences in item score change (red bars) across the MADRS, HRS, BDI_IA_, and QIDS-SR_16_ scales using item scores computed on the same response scale. Items most favorable to PT included *MADRS Reported Sadness* (*B* = −0.20), *MADRS Lassitude* (*B* = −0.18), *HRS Libido* (*B* = −0.38), *HRS Somatic energy* (*B* = −0.21), *HRS Work and interests* (*B* = −0.18), *HRS Agitation* (*B* = −0.18), *BDI Guilt* (*B* = −0.23), *BDI Dissatisfaction with life* (*B* = −0.19), *BDI Reduced sexual interest* (*B* = −0.19), and *BDI Worthlessness* (*B* = −0.16).

These results suggest that energy level, self-appraisal, amotivation (with specific emphasis on libido), and anhedonia are symptom domains that especially favor the action of PT over ET.

Although QIDS-SR_16_ contained some of these facets (e.g., energy level, restlessness), most are absent from the QIDS-SR_16_, namely guilt, anhedonia, libido, and perceived attractiveness. In addition, it bears noting that all of the QIDS-SR_16_ items most differentially responsive to PT, including *Falling asleep* (*B* = −0.15), *Sleeping too much* (*B* = −0.11), *Feeling slowed down* (*B* = −0.08), *Feeling restless* (*B* = −0.08), were subsumed within compound criteria such that patients’ scores on these items were not necessarily reflected in their sum-scores. That is, differential response in these items was masked by combining them with other less differentially responsive items within compound criteria, for example, *Falling asleep* (*B* = −0.15) and *Sleeping too much* (*B* = −0.11) were combined with *Sleep during the night* (*B* = 0.05) and *Waking up too early* (*B* = −0.08) to make up the *Sleep compound criterion*. Furthermore, only the highest-scored item among these four was selected, meaning that differentially responsive items like *Falling asleep* were not reflected within many patients’ sum-scores.

#### Examining between-condition differences in item-level change

**Figure 3.**
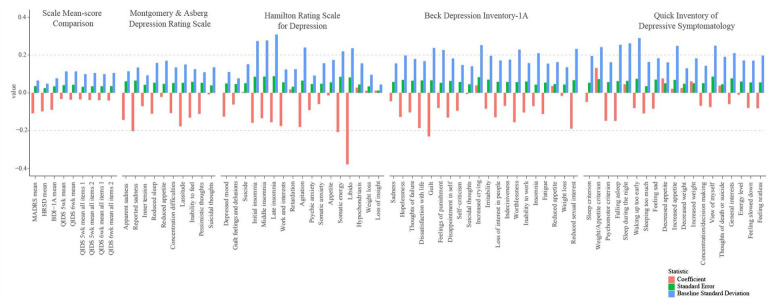
Scale-level comparison.

To assess the validity of the QIDS-SR_16_ using data from Carhart-Harris et al. (2021), QIDS-SR_16_ items were compared with similar items from other scales that would be predicted to show a similar pattern of differential treatment response. Where discrepancies were found between QIDS-SR_16_ items and items from other scales, a rational analysis of item content was undertaken to identify the source of the discrepancy. Figure 3 illustrates estimates of between-condition differences in item score change across 11 areas of depression including depressed mood (instantiated in *QIDS Feeling sad*), amotivation/interests (*QIDS General interests*), negative self-appraisal (*QIDS View of myself*), energy level (*QIDS energy level*), concentration/indecisiveness (*QIDS Concentration/decision making*), suicidal thoughts (*QIDS Thoughts of death and suicide*), insomnia (*QIDS Falling asleep*, *Sleep during the night*, *Waking up too early*, *Sleeping too much*), reduced weight/appetite (*QIDS Decreased appetite*, *Decreased weight*), psychomotor retardation (*QIDS Feeling slowed down*), and psychomotor restlessness (*QIDS Feeling restless*).

##### Evidence of differences in QIDS-SR_16_ item functioning

With respect to negative self-appraisal, the QIDS-SR_16_ appeared less responsive to relevant between-condition changes. *QIDS View of myself* exhibited a lower between-condition difference (*B*_6wks_ = −0.07) compared with all other scale items with similar content, except for *HRS Guilt feelings and delusions.* Three observations were notable. First, *QIDS View of myself* is a compound item containing multiple symptoms of negative self-appraisal within it (e.g., worthlessness, guilt, self-criticism) whose broadness may fail to adequately measure clinically relevant individual symptoms of self-appraisal. By contrast, the BDI_IA_ measured negative self-appraisal using narrow items that indexed individual symptoms including *BDI Guilt* (*B* = −0.23), *Worthlessness* (*B* = −0.16; reflecting perceptions of attractiveness), and *Disappointment in self* (*B* = −0.13). Second, BDI_IA_ notably contains a higher proportion of items indexing negative self-appraisal (BDI_IA_ = 24%, QIDS = 11%). To the degree that negative self-appraisal is differentially responsive to the present treatments, this property may account for differences in results between the BDI_IA_ and QIDS-SR_16_ sum-scores. Third, it is not clear that the 0–3 response options for *QIDS View of Myself* follow an ordinal scheme, for example, a score of “3” for this item reads “I think almost constantly about major and minor defects in myself,” a score of “2” reads “I largely believe that I cause problems for others,” a score of “1” reads: “I am more self-blaming than usual.” Lack of appropriate ordinality was psychometrically reflected in a large sample (*N* = 2542) of healthy prospective psychedelic users from the general population who exhibited the following pattern of responses at baseline assessment (0: *N* = 1518, 1: *N* = 532, 2: *N* = 100, 3: *N* = 393; Kettner et al., 2021; Weiss et al., 2021). With ordinality, in the normal population one would expect a lower rate of endorsement as symptom severity increases. These finer grain issues are perhaps best appreciated by viewing the QIDS-SR_16_ items and score choices themselves (Supplemental Table S8).

With respect to energy level, the QIDS-SR_16_ showed anomalous performance relative to MADRS, HRS, and BDI_IA_. Whereas *QIDS Energy level* exhibited a between-condition difference of −0.05 and −0.01 at 5 and 6 weeks, respectively, MADRS, HRS, and BDI_IA_ items with similar content exhibited substantially higher effect sizes in the same direction. Notably, *MADRS Lassitude* (*B* = −0.18), *HRS Somatic energy* (*B* = −0.21), and *HRS Work and interests* (*B* = −0.18) were among the most favorable to PT. Part of this difference may emanate from differences between self-report scales and clinician-rated scales. Whereas clinician-rated scales assess patients’ relative difference from normal/healthy functioning, self-report measures rely on patients’ own evaluation for this comparison (e.g., “There is no change in my usual level of energy” *QIDS Energy level*). To the degree that patients have experienced longstanding low energy level and compare their current energy level to this already elevated benchmark, they may be more likely to select a low response choice. However, it is not clear how much this property contributed to differences between self-report and clinician-ratings, and this property cannot account for differences between QIDS-SR_16_ and BDI_IA_, which similarly relies on patients’ assessment of their “usual” level.

We observed two possible reasons for the discrepant QIDS performance for energy levels relative to the BDI_IA_. First, whereas *BDI Inability to work* and *Fatigue* items contained respective response choices that homogenously indexed each symptom, *QIDS Energy level* was compound, containing one general energy level response choice, one fatigue response choice, and two work-related response choices. The compound nature of this item may drive differences in interpretation and mask clinically relevant changes in symptoms not being considered or interpreted by the respondent. Second, *QIDS Energy level* differed from the comparable BDI_IA_ item *Inability to work* in being more specific with respect to functional work-related behaviors. For example, the QIDS-SR_16_ contains a response choice containing “I have to make a big effort to start or finish my usual daily activities (for example, shopping, homework, cooking, or going to work),” whereas the BDI_IA_ contains the following response choice: “I have to push myself very hard to do anything.” In sum, BDI response choices were more symptom homogeneous and precise.

With respect to suicidality, curiously, *QIDS Thoughts of death and suicide* showed a between-condition effect in the opposite direction to *MADRS Suicidal thoughts* (*B*_6wks_ = −0.01) and *BDI Suicidal thoughts* (*B*_6wks_ = −0.01), though these estimates are unlikely to be substantively different. The largest content-level difference between *QIDS Thoughts of death* and the other items is the QIDS’ allusion to “death” in addition to suicide, which may lead patients to endorse the item in the absence of suicidality, but rather in the presence of thoughts of mortality, which may be elevated following psychedelic experience—and in a non-dysphoric way (Timmermann et al., 2018).

With respect to sleep, the QIDS-SR_16_ showed a different pattern of functioning compared to items with similar content in two respects. On one hand, *QIDS Waking up too early* (*B*_6wks_ = −0.08) showed a comparable effect size and pattern compared to *BDI Insomnia* (*B*_6wks_ = −0.07). This similarity is understandable given that *BDI Insomnia* is a compound item that devotes two of four of its response choices to late insomnia (i.e., waking up early). On the other hand, *QIDS Sleep during the night* (*B*_6wks_ = 0.05) showed a pattern that markedly differed from *BDI Insomnia* (*B* = −0.07) and *HRS Middle insomnia* (*B* = −0.13), namely a small effect size in the opposite direction, favoring ET. This sizable difference of opposite direction is difficult to reconcile. Of possible pertinence is the *QIDS Sleep during the night* item’s inclusion of behaviorally specific content focused on waking (e.g., “I awaken more than once a night and stay awake for 20 minutes or more, more than half the time”), whereas the HRS item invites the clinician to rate any of multiple components of middle insomnia (e.g., restlessness, disturbance, waking). In addition, comparing QIDS Sleep items to the *QIDS Sleep criterion* reveals a possible masking effect. Whereas *QIDS Sleep criterion* showed a small between-condition difference favorable to PT (*B*_6wks_ = −0.05), *QIDS Falling asleep* (*B*_6wks_ = −0.15) and *QIDS Sleeping too much* (*B*_6wks_ = −0.11) showed substantial effects favorable to PT. This pattern may be suggestive that the QIDS’ compound construction of the *Sleep criterion* may serve to mask the differential effects of the present treatments on particular Sleep-related individual symptoms that showed markedly mixed results.

With respect to weight/appetite, QIDS-SR_16_ showed a pattern of between-condition differences more strongly favorable to ET. The *QIDS Weight/Appetite criterion* in particular showed a between-condition difference favoring escitalopram (*B*_6wks_ = 0.13). By contrast, MADRS, HRS, and BDI_IA_ items with similar content showed small, mixed effects. *QIDS Weight/Appetite* criterion’s effect may account in part for the QIDS-SR_16_’s differential sum-scale results relative to other scales.

With respect to psychomotor retardation, *QIDS Feeling slowed down* (*B*_6wks_ = −0.08) differed from comparable items (i.e., *HRS Retardation*, *B* = 0.02) in showing a between-condition difference favorable to PT. A major difference between these two items is that *HRS Retardation* involves assessment of retardation during the clinical interview, whereas *QIDS Feeling slowed down* relies on patients’ self-appraisal.

With respect to psychomotor restlessness, the *QIDS Feeling restless* (*B*_6wks_ = −0.08) exhibited a smaller between-condition difference than *HRS Agitation* (*B* = −0.18), though both items favored PT. A major difference between these two items is that *HRS Agitation* involves assessment of restlessness during the clinical interview, whereas *QIDS Feeling restless* relies on patients’ self-appraisal.

##### Evidence of mixed results

With respect to amotivation/interests, the QIDS-SR_16_ showed mixed results. At 5 weeks, the *QIDS General interests* (*B* = −0.15) showed a between-condition difference comparable to BDI_IA_ items with similar item content (e.g., *BDI Loss of interest in people: B* = −0.13; *BDI Reduced sexual interest: B* = −0.19). However, at 6 weeks, the *QIDS General interests* (*B* = −0.06) showed an effect size substantively lower than comparable BDI_IA_ items. The pattern of QIDS results could be suggestive that scores became less favorable to PT between week 5 to week 6, and that BDI_IA_ scores at week 6 merely reflect patients’ depression at week 5. However, because it seems unlikely that patients completing the BDI_IA_ would differentially weight symptoms in week 5 versus week 6, it is plausible that psychometric differences between *QIDS General interests* at week 6 and the BDI_IA_’s comparable items at week 6 account for the discrepancy. We therefore ventured to interpret the possible reasons for a discrepancy at week 6, observing two tentative reasons for aberrant QIDS functioning.

First, *QIDS General interests* is compound in its response options and focus. The item asks patients about their interest in people and activities in two lower severity response options, but only references people in the two higher severity response choices. In contrast, *BDI Loss of interest* in people asks about people in all response choices. It is conceivable that focusing on interest in activities versus people in the QIDS masks a stronger differential effect of treatment on interest in people particularly.

Second, given the discrepancy in scores on *BDI Reduced sexual interest* versus *QIDS General interests*, it seems plausible that respondents to the *QIDS General interests* did not interpret the item in such a way that sexual interest/activity was considered. Given the apparent responsiveness of sexual amotivation to PT versus ET, such a pattern of interpretation would limit the QIDS-SR_16_ from detecting change in this symptom of depression.

Third, consistent with the second point, anhedonia is not represented among the QIDS-SR_16_ measures. Given the substantive differential response observed in *BDI Dissatisfaction with life*, it is possible that the QIDS-SR_16_ merely excludes symptoms that are particularly differentially responsive to the present treatments. However, given the relatively comparable differential response in *QIDS General interests* at 5 weeks, these explanations of discrepancy between the QIDS-SR_16_ and other scales remains tentative.

##### Evidence of no substantive differences in QIDS-SR_16_ item functioning

With respect to depressed mood, *QIDS Feeling sad* (*B*_6wks_ = −0.08) showed a between-condition difference comparable to BDI_IA_ self-report items with similar content (e.g., *BDI Sadness: B* = −0.04), but a lower effect compared with clinician-rated measures (e.g., *MADRS Reported sadness: B* = −0.20).

With respect to concentration and indecisiveness, *QIDS Concentration/decision making* (*B*_6wks_ = −0.07) appeared to function comparably to other self-report items with similar content (e.g., *BDI Indecisiveness: B* = −0.07).

### Examining compound criteria

The extent to which QIDS-SR_16_ compound criteria contributed to measurement error was examined, through observing the number of participants who scored different compound criterion items at baseline and 6 weeks. Table 2 shows the specific item changes among these patients and the item and item change score correlations for each pair of items. For the *Sleep criterion*, 13 patients (22%) exhibited inconsistency in which Sleep item was scored highest across the two timepoints. For the *Weight criterion*, 11 patients (19%) exhibited inconsistency in which Weight item was scored highest across the two timepoints. Lastly, for the *Psychomotor criterion*, four patients (7%) exhibited inconsistency across timepoints. Table 2 also illustrates the intercorrelations between the pairs of different highest-scored items. Relations between pairs varied widely and largely failed to show moderate-to-large baseline intercorrelation and covariation over time.

**Table 2.** Examining specific cases of inconsistency in highest-scored items across timepoints.

Inconsistency pattern	# patients (%)	*r* Δitem	*r* item
Sleep			
QIDS2 → QIDS3	2 (3)	0.05	0.24
QIDS3 → QIDS2	4 (7)	0.05	0.24
QIDS1 → QIDS2	2 (3)	0.00	−0.04
QIDS2 → QIDS1	5 (8)	0.00	−0.04
Weight/appetite			
QIDS6 → QIDS7	1 (2)	−0.22	−0.31
QIDS7 → QIDS6	2 (3)	−0.22	−0.31
QIDS6 → QIDS8	1 (2)	0.52	0.33
QIDS7 → QIDS8	3 (5)	−0.12	−0.27
QIDS7 → QIDS9	3 (5)	0.54	0.65
QIDS8 → QIDS9	1 (2)	−0.27	−0.36
Psychomotor			
QIDS15 → QIDS16	4 (7)	0.02	−0.03

Each row indicates a pattern of responding in which a patient scores one item within each compound criterion highest at baseline and a different one at 6 weeks, creating inconsistency. The “# patients (%)” column indicates the number of patients who exhibited the pattern under the first column. “*r* Δitem” indicates the correlation between change in the first item and change in the second item between baseline and week 6; “*r* item” indicates the correlation between the two items at baseline.

Two different computations of the *QIDS-SR_16_ mean-score* were conducted in which the highest item score selection operation was omitted. The first computation included all items except for *QIDS Sleep too much*, *QIDS Increased appetite*, and *QIDS Increased weight* (*QIDS mean all items 1*). The second computation included all items except for *QIDS Sleep too much*, *QIDS Decreased appetite*, and *QIDS Decreased weight* (*QIDS mean all items 2*). When compared to the normal QIDS-SR_16_ sum-score on the same response scale, the between-condition difference estimate changed marginally (i.e., *QIDS mean all items 1*: Δ*B* = −0.12; *QIDS mean all items 2*: Δ*B* = −0.08), while the standard error decreased by 18% (*QIDS mean all items 1*) and 17% (*QIDS mean all items 2*).

### Comparison of standard error and variance

Differences in standard error, baseline variance, and change score variance across depression scales were examined to potentially account for null between-condition results respecting QIDS-SR_16_. Table 3 and Figure 2 presents the between-condition difference standard error and baseline variance, and change score variance for the MADRS, HRS, BDI_IA_, and QIDS-SR_16_ mean-scores with scores computed on the same response scale. Standard error, baseline variance, and change score variance were larger for the QIDS-SR_16_ than all other scales. Specifically, the standard error for the QIDS-SR_16_ between-condition interaction coefficient was 19% higher than the *BDI_IA_ mean-score*’s standard error, 21% higher than the *MADRS mean-score*’s standard error, and 76% higher than the *HRS mean-score*’s standard error. The standard deviation of baseline *QIDS-SR_16_ mean-score* was a substantial 47% higher than the BDI_IA_, 74% higher than the MADRS, and a remarkable 135% higher than the HRS. Finally, the standard deviation of change in *QIDS-SR_16_ mean-score* between baseline and 6 weeks was 11% higher than the BDI_IA_, 14% higher than the MADRS, and 58% higher than the HRS. These indications of higher variance for the QIDS-SR_16_ could be reflective of higher measurement error.

**Table 3.** Examining the standard error and variance of depression scale scores.

Scale score	Standard error	Baseline standard deviation	Change score standard deviation
MADRS mean-score	0.04	0.07	0.14
HRS mean-score	0.02	0.05	0.10
BDI_IA_ mean-score	0.03	0.08	0.14
QIDS-SR-16 mean-score 5 weeks	0.04	0.11	0.15
QIDS-SR-16 mean-score 6 weeks	0.04	0.11	0.16
QIDS all items 1 5 weeks	0.03	0.10	0.12
QIDS all items 2 5 weeks	0.03	0.11	0.13
QIDS all items 1 6 weeks	0.03	0.10	0.13
QIDS all items 2 6 weeks	0.04	0.11	0.14

QIDS all items 1 and 2 represent QIDS mean-score composites. Standard error reflects the standard error of the interaction term coefficient in linear mixed effects models in which mean-score is regressed onto *Time* *×* *Condition*.

BDI_IA_: Beck Depression Inventory-IA; HRS: Hamilton Rating Scale for Depression; MADRS: Montgomery and Asberg Depression Rating Scale; QIDS-SR-16: Quick Inventory of Depression Symptoms-Self-report.

### Reexamining the efficacy of PT versus ET using two inclusive approaches

#### Depression facets across five depression and anhedonia scales

In view of potential psychometric problems with the QIDS-SR_16_ and the HRS’ poor internal consistency, a second approach was undertaken in which items from all four depression scales and one anhedonia scale were used to derive seven depression facet outcomes based on Ballard et al.’s (2018) factor structure. The motivation was to identify core components (or facets) of depression across rating scales.

Specifically, using LME models, we examined the differential efficacy of PT versus ET on these depression facet scores. A *Condition* × *Time* interaction explained a large amount of variance in *Depressed mood* and *Anhedonia*, with results indicating significant moderation of change in *Depressed mood* (*B*_int_ = −0.11, *b*_int_ = −0.68, *p* = 0.013) and *Anhedonia* (*B*_int_ = −0.12, *b*_int_ = −0.79, *p* = 0.001) by *Condition*.

More specifically, contrasting baseline to the 6 week endpoint, these results show that the PT condition was associated with a greater reduction in *Depressed mood* by 0.68 standard deviations and *Anhedonia* by 0.79 standard deviations, relative to the ET condition.

**Figure 4.**
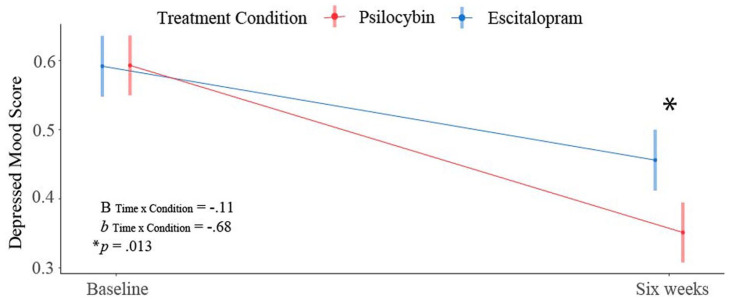
Plot illustrating stronger response in the depressed mood facet (based on Ballard et al.’s (2018) factor structure) in the PT arm versus the ET arm. Although patients in both groups exhibited the same initial level of depressed mood, patients in the PT arm reported a greater reduction in symptom severity (*p* = 0.013). *b*: standardized *Time* × *Condition* interaction term; *B*: unstandardized *Time* × *Condition* interaction term.

Figure 4 shows a graphical depiction of this pattern, which was shared across the two facets. Significant condition differences were not observed in the other domains. Results for *Depressed mood* and *Anhedonia* can be found in Table 4. Full results can be found in Supplemental Table S6.

**Table 4.** Examining between-condition differences in *Depressed mood*, *Anhedonia*, and *Depression Factor*.

		*b*	SE (*b*)	*B*	SE (*B*)	DF	*t*-Value	*p*-Value
*Depressed mood*	(Intercept)	0.60**	0.14	0.59	0.02	112	4.25	<0.001
	*Condition*	0.01	0.20	0.00	0.03	112	0.04	0.970
	*Time*	−0.87**	0.19	−0.14	0.03	57	−4.63	<0.001
	*Time × Condition*	−0.68*	0.26	−0.11	0.04	57	−2.57	0.013
*Anhedonia*	(Intercept)	0.43**	0.16	0.62	0.02	90	25.80	<0.001
	*Condition*	0.08	0.22	0.01	0.03	90	0.38	0.705
	*Time*	−0.55**	0.16	−0.08	0.02	57	−3.49	0.001
	*Time × Condition*	−0.79**	0.22	−0.12	0.03	57	−3.61	0.001
*Depression Factor*	(Intercept)	0.63**	0.15	0.65	0.02	108	4.30	<0.001
	*Condition*	−0.06	0.20	−0.01	0.03	108	−0.30	0.764
	*Time*	−0.91**	0.18	−0.15	0.03	57	−5.08	<0.001
	*Time × Condition*	−0.55*	0.25	−0.09	0.04	57	−2.17	0.035

“Intercept” reflects mean outcome estimate at baseline for ET arm patients; “*Condition*” reflects the effect of condition on outcome at baseline; “*Time*” reflects the difference between conditions in outcome scores for the ET condition; “*Time × Condition*” reflects the difference between conditions in changes in outcome scores between baseline and 6 weeks.

*b*: standardized coefficient; *B*: unstandardized coefficient.

* *p* < 0.05. **p < 0.01.

#### Single factor across four depression scales

In the second approach, we examined the effect of PT versus ET on the *Depression Factor* score that emerged from a factor analysis on all 64 items from the four aforementioned depression rating scales, that is, this identified 15 items and item-composites from a mix of rating scales that each loaded above 0.40 onto the factor. The motivation was to identify a core factor of depression. These 15 items/composites can be viewed in Table 5, and full factor loadings can be found in Supplemental Table S4.

**Table 5.** Items and item-composites comprising the *Depression Factor* score.

Item	λ	Communality	Uniqueness
BDI Item 1 Sadness	0.69	0.48	0.52
QIDS Depressed Mood	0.69	0.48	0.52
BDI Amotivation	0.68	0.47	0.53
QIDS Item 11 View of Myself	0.67	0.45	0.55
BDI Negative Cognition	0.59	0.34	0.66
BDI Item 3 Thoughts of Failure	0.56	0.32	0.68
QIDS Impaired Sleep	0.54	0.29	0.71
QIDS Item 13 General interests	0.52	0.27	0.73
MADRS Depressed Mood	0.51	0.26	0.74
QIDS Item 10 Concentration/Decision-making	0.51	0.26	0.74
BDI2 Hopelessness	0.49	0.24	0.76
MADRS9 Pessimistic Thoughts	0.48	0.23	0.77
MADRS Item 3 Inner Tension	0.47	0.22	0.78
HRS Depressed Mood	0.44	0.19	0.81
QIDS Item 1 Falling Asleep	0.41	0.17	0.83

BDI: Beck Depression Inventory; HRS: Hamilton Rating Scale for Depression; MADRS: Montgomery and Asberg Depression Rating Scale; QIDS: Quick Inventory of Depressive Symptomatology.

QIDS Depressed Mood contains QIDS5 (*Feeling sad*) and QIDS15 (*Feeling slowed down*). BDI Amotivation contains BDI4 (*Dissatisfaction with life*), BDI11 (*Irritability*), BDI12 (*Loss of interest in people*), BDI13 (*Indecisiveness*), and BDI15 (*Inability to work*). BDI Negative Cognition contains BDI10 (*Increased crying*), BDI5 (*Guilt*), BDI6 (*Feelings of punishment*), and BDI7 (*Disappointment in self*). QIDS Impaired Sleep contains QIDS2 (*Sleep during the night*) and QIDS3 (*Waking up too early*). HRS Depressed Mood contains HAMD1 (*Depressed mood*), HAMD7 (*Work and interests*), and HAMD8 (*Retardation*); MADRS Depressed Mood contains MADRS1 (*Apparent sadness*), MADRS2 (*Reported sadness*), MADRS6 (*Concentration difficulties*), and MADRS8 (*Inability to feel*).

Importantly, results indicated significant moderation of change in the *Depression Factor* by condition (*B*_int_ = −0.09, *b*_int_ = −0.55, *p* = 0.035). Being in the PT condition was associated with a greater reduction in depression by .55 standard deviations, relative to the ET condition. The pattern of this change is similar to that displayed in Figure 4, and full results are provided in Table 4.

## Discussion

The present study explored the psychometric validity of QIDS-SR_16_ using data from a trial of PT versus ET for depression. As highlighted in the original trial report (Carhart-Harris et al., 2021), the QIDS-SR_16_ differed from other efficacy rating scales in not exhibiting a treatment response favoring PT versus ET. Here we endeavored to resolve the discrepancies between the QIDS-SR_16_ and other scales in an effort to understand this anomalous result.

### What accounts for the discrepancy between the QIDS-SR_16_ and other depression scales?

Evidence for the discrepancy between the QIDS-SR_16_ and other depression scales was multi-factorial. Possible factors included higher variance and standard error in QIDS-SR_16_ scores (which could reflect measurement imprecision), lower sensitivity of particular QIDS-SR_16_ items due to compound item properties, differences in the weighting of depression symptoms/facets that are differentially responsive to PT (e.g., a lack of items related to negative cognition in the QIDS-SR_16_), and mixed patterns of differential response across QIDS-SR_16_ items (e.g., among Sleep items) that may have masked the effects of symptoms/facets differentially sensitive to PT or ET.

Perhaps the strongest evidence for the discrepancy between the QIDS-SR_16_ and other depression scales emerged from a rational analysis of QIDS items that showed a different pattern of differential response when comparing similar items across scales. Although QIDS-SR_16_ items functioned comparably to similar items from other scales with respect to certain symptom domains, including depressed mood and concentration/indecisiveness, on domains including energy level, amotivation, negative self-appraisal, QIDS-SR_16_ items showed markedly lower treatment response.

A rational analysis of item content raised possibilities that certain QIDS-SR_16_ items are insensitive to differential response as a result of enquiring about symptoms in a manner that was too variegated and imprecise (e.g., as in the case of *QIDS View of myself*), or including items that contain compound symptoms within that item (as in *QIDS General interests* and *QIDS Energy level*). Moreover, the wording of the 0–3 categories for certain items such as *QIDS View of Myself* do not always intuitively follow an ordinal scheme. These finer-grain issues are perhaps best appreciated by viewing the QIDS-SR_16_ items and response options themselves (Supplemental Table S8).

The QIDS-SR_16_ was also observed to neglect symptoms showing higher responsiveness to PT versus ET. For example, a lower overall proportion of narrow self-appraisal symptoms was observed. Although the BDI_IA_ has been criticized for weighting cognitive symptoms more heavily (Hagen, 2007; Rush et al., 1986), subsequent research has shown that such symptoms bear strong clinical relevance when compared to DSM-instantiated symptoms such as sleep, weight/appetite, and psychomotor dysfunction (Fried and Nesse, 2014; Fried et al., 2016a). Moreover, symptoms bearing highest responsiveness to PT including anhedonia, guilt, sexual dysfunction, and perceived attractiveness were not as well represented in the QIDS-SR_16_.

Finally, the QIDS-SR_16_ was unique among measures in showing numerically differential response favoring ET in weight/appetite problems and suicidality. Although not statistically significant, this pattern could have contributed toward masking true differential treatment efficacy between PT versus ET, that is, when interpreting results via an undifferentiated sum-score.

Our examination of measurement error showed substantive, but weaker evidence of problematic QIDS-SR_16_ functioning. First, substantial differential treatment responses in *QIDS Falling asleep* and *QIDS Sleeping too much* showed evidence of being obscured by the use of the compound *QIDS-SR_16_ Sleep criterion*, an issue illustrating relative imprecision in the QIDS-SR_16._ However, excluding compound items from the *QIDS-SR_16_ mean-score* did not meaningfully alter differential response estimates. Therefore, it is not likely that the compound criteria used in the QIDS-SR_16_ can fully account for the discrepancy between scales. Second, the QIDS-SR_16_ mean-score exhibited substantively higher variance in baseline and change scores than other scale mean-scores. However, this property cannot be straightforwardly interpreted. The QIDS-SR_16_’s greater proportion of compound items, and the observed trend of decreased variance when eliminating compound criteria may be suggestive, but not definitively indicative, of measurement error. Third, inconsistency in the highest-scored item between baseline and 6 weeks was observed for the QIDS-SR_16_ sleep criterion and the weight/appetite criterion in 22 and 19% of patients, respectively, and small (and sometimes negative) intercorrelations between the relevant item pairs indicated that these items did not show adequate evidence of indexing the same construct.^2^

On balance, these results raise concerns about the precision of certain QIDS-SR_16_ items for detecting differential treatment response. In general, the pattern of results is suggestive that the use of certain compound items and scale sum-scores, more broadly, may obfuscate the signal-to-noise ratio in differential treatment response. These results also provide further empirical support to, in our view, compelling calls for measurement of individual symptoms and facets of depression (Fried and Nesse, 2015) in view of lack of unidimensionality within the depression construct (Ballard et al., 2018; Fried et al., 2016b; Shafer, 2006), substantial differences in content across measures of depression (Fried, 2017), and differential treatment response from symptoms (Hieronymus et al., 2016a).

### Understanding differential treatment response at the item, facet, and single factor level

One of the most important contributions of the present research is its identification of symptoms and facets of depression most responsive to PT versus ET. *Item-level* results were indicative of particularly strong differential changes in symptoms related to the positive valence system (i.e., amotivation, anhedonia, energy level, perceived attractiveness) and negative valence system (i.e., guilt)—all of which favored PT.

Of note, detection of differential response in sexual interest (or libido) would not have been possible outside of item-level analysis, and this result was present across self-report and clinician-rated scales. Response in this symptom may be particularly important given robust evidence of treatment-emergent sexual dysfunction related to escitalopram and SSRIs more broadly (Cascade et al., 2009; Clayton et al., 2007). Given the importance of sexual functioning to well-being and relationship satisfaction (Heiman et al., 2011; Laumann et al., 1999), as well as the relevance of libido to amotivation and anhedonia, PT’s superiority over SSRI pharmacotherapy in remediating this domain is important, especially among patients who regard sexual dysfunction as particularly impairing.

More broadly, it may be instructive that the symptom areas most responsive to PT involve a reallocation of energy to involvement with valued people and activities, including sexual functioning. The analytical rumination hypothesis (Andrews and Thomson, 2009), which shares similarities with Sigmund Freud’s theory of depression (Carhart-Harris et al., 2008), holds that a depressed state is a preserved evolutionary adaptation by which humans, faced with complex social dilemmas, internalize metabolic resources, diverting them onto ruminative problem solving, thereby depleting reserves that would otherwise be invested into biological or external imperatives such as sleep, sustenance, sex, and communality. Evidence for greater capacity to deploy metabolic resources elsewhere (e.g., energy, interest) after PT may exemplify its relative therapeutic value.

*Facet-level* results were indicative of differential treatment response favoring PT in depressed mood and anhedonia, specifically, but not in amotivation, negative cognition, reduced appetite, impaired sleep, or suicidal thoughts.^3^ Notably, anhedonia is *not* well represented in the QIDS-SR_16_.

Compared with other symptoms of depression, depressed mood and anhedonia are particularly clinically relevant as they are among the most causally central to the network of depression symptoms (Fried et al., 2016a) and bear strong relations to psychosocial impairment (Fried and Nesse, 2014). These results are therefore suggestive that PT may be superior to ET in addressing core aspects of depression involving negative and positive emotion. This possibility may help inspire the discovery of core biomarkers related to a hypothesized core dimension of depression. Replicated decreases in whole-brain modularity could be a candidate in this regard (Daws and Carhart-Harris, 2022). One might also note that this recent fMRI result resonates with treatment mechanisms intuited by recent authors as being relevant to depression, namely “attractor dynamics” in depression and their targeting by effective treatments (Fried and Robinaugh, 2020; Fried et al., 2022; Olthof et al., 2020).

These facet-level differential responses were present even when controlling for relative expectancy, strengthening the inferences we can draw on direct treatment effects of PT versus, for example, a placebo-related action (Szigeti et al., 2022). Conversely, these results are suggestive that PT and SSRI therapies may be equivalent with respect to other facets of depression, most notably reduced appetite and suicidality (although note the SIDAS result in Carhart-Harris et al., 2021).

Results were additionally indicative of differential treatment response in the *EFA-derived single depression factor*. This factor was comprised of core symptoms of depression that best explained variance in all symptoms measured across the four depression scales. These core symptoms tended to reflect facets of depressed mood, negative self-appraisal, and amotivation. This supplementary finding is notable for, on this occasion, including the domains of amotivation and negative cognition (i.e., self-appraisal).

Perhaps the most consistent result across levels of analysis was differential change in depressed mood. This is notable because network models of depression have consistently identified depressed mood as a symptom with strongest links to other symptoms (Beard et al., 2016; Fried et al., 2016a), meaning that this symptom may be a causal linchpin in subsequent cascades of depressive symptoms. Depressed mood has also been observed to bear strongest association to psychosocial impairment when compared with other symptoms (Fried and Nesse, 2014). Therefore, remediation of depressed mood may be pivotal in modulating depressive symptomology and impairment.

### Recommendations for depression measurement

The larger implication of this work is that analyzing change using whole scale sum-scores, that do not (and should) break down scales into more orthogonal factors, can function to mask true and important factor- or facet-level and symptom-level changes that could, for example, differentiate the efficacy of different treatments with different mechanisms of action. Accordingly, inclusive approaches that derive outcomes at the symptom- and facet-levels of analysis, as done here, are likely to be more sensitive in detecting clinically useful treatment differences. We accordingly support the development of scales that index core and facet-level depression standing, as well as a priori designs that pre-specify particular core and facet composites from items spanning multiple scales. Consistent with other scientists (Cuijpers et al., 2010), we recommend combining self- and clinician-ratings, which possess unique benefits and costs (see Supplemental Materials III for further discussion). Finally, if pressured to recommend particular scales, the present results provide support for the BDI_1A_ (or subsequent versions) and HRS as self-report and clinician-rated instruments, respectively, with greater sensitivity, lower measurement error, and superior symptom coverage.

### Limitations

Some limitations of the present work should be noted. First, although patient expectancy was controlled for in the present analyses, the expectancies of clinicians and other rating biases were not measured and could not be controlled for. Second, the facet-level examination of differential treatment response was based on Ballard et al.’s (2018) factor structure of depression. This EFA-derived factor structure was originally based on relatively low sample size (*N* = 119), and has not been replicated using confirmatory methods. Therefore, the results of these analyses are accordingly tentative. Third, post hoc analyses on data with small sample size risks type I error, that is, false positives. Results from the present analyses should therefore be considered exploratory and dependent on future replication. Fourth, conclusions regarding the psychometric weaknesses of the QIDS-SR_16_ should be moderated in proportion to the small sample size used here as well as the specificity of the research area under examination. Fifth, although we attempted to gauge measurement error by reference to variance and standard error in the data, measurement error cannot be definitively ascertained by these properties, and our estimates could equally emanate from greater precision in the QIDS-SR_16_ for reflecting population variance.

## Conclusion

Multiple sources may have contributed to the discrepant findings on the QIDS-SR_16_ in *A Trial of Psilocybin versus Escitalopram for Depression* (Carhart-Harris et al., 2021). Chief among these are (1) higher variance on the QIDS-SR_16_; (2) its imprecision due to compound items; (3) whole-scale, unidimensional sum scoring; (4) its lack of focus on a core depression factor; and (5) vagueness in the phrasing of scoring options for individual items—creating data that may at times be more ordinal than nominal.

Evidence of plausible sources of insensitivity on the QIDS-SR_16_ led us to re-analyze the trial data at an item-, facet-, and factor-level. This approach yielded important information about symptoms and facets of depression that are differentially responsive to PT versus ET and thus, have a bearing on how the original trial findings of *A Trial of Psilocybin versus Escitalopram* might be interpreted. At the item-level, a treatment difference in changes in libido was observed, signaling a potential key advantage of PT therapy in avoiding onerous SSRI-related side effects involving sexual dysfunction. At the facet-level, depressed mood and anhedonia emerged as differentially responsive, whereas others did not. Should these results replicate in future work, this could be indicative that PT is superior to ET in addressing two of the most causally central and psychosocially impairing symptoms of depression.

## Supplemental Material

sj-docx-1-jop-10.1177_02698811231167848 – Supplemental material for A critical evaluation of QIDS-SR-16 using data from a trial of psilocybin therapy versus escitalopram treatment for depression Click here for additional data file. Supplemental material, sj-docx-1-jop-10.1177_02698811231167848 for A critical evaluation of QIDS-SR-16 using data from a trial of psilocybin therapy versus escitalopram treatment for depression by Brandon Weiss, David Erritzoe, Bruna Giribaldi, David J Nutt and Robin L Carhart-Harris in Journal of Psychopharmacology

## Notes

1. Sample size was 57 and not 59 because two patients were missing data for the Montgomery and Asberg Depression Rating Scale and Hamilton Rating Scale for Depression.

2. It should be acknowledged, however, that intercorrelation estimates from this sample differed from larger samples in which weak to moderate correlations between baseline item scores were more typical (e.g., Fried et al., 2016b)

3. Nevertheless, a specific suicidality scale significantly favored PT in the first set of published analyses (Carhart-Harris et al., 2021).
