# Supplementary material for: A critical evaluation of QIDS-SR-16 using data from a trial of psilocybin therapy versus escitalopram treatment for depression
Source: J Psychopharmacol. 2023 Apr 25;37(7):717–32. doi: 10.1177/02698811231167848 (PMC10350722; doi:10.1177/02698811231167848)
Supplement: sj-docx-1-jop-10.1177_02698811231167848 – Supplemental material for A critical evaluation of QIDS-SR-16 using data from a trial of psilocybin therapy versus escitalopram treatment for depression [file sj-docx-1-jop-10.1177_02698811231167848.docx]

A critical evaluation of QIDS-SR-16 using data from a trial of psilocybin vs escitalopram for depression

**Supplementary Materials**

**Supplementary Materials I: Construct validity of granular domains**

To demonstrate the construct validity of the granular depression factors, associations among factor scores (Table S2), and associations between factor scores and convergent constructs (Table S5) were tested. Results indicated substantial overlap among Amotivation, Negative Cognition, and Depressed Mood; moderate overlap between Anhedonia and these factors; moderate overlap between Suicidality and Depressed Mood; and minimal overlap between Reduced Appetite and Impaired Sleep, on the one hand, and all other factors, on the other. Tests of convergent validity indicated that, congruent with hypotheses, Negative Cognition and Depressed Mood were correlated (*p*<.05) with Big Five Inventory-I (BFI; John, Donahue, & Kentle, 1991) Neuroticism (positively) and Rosenberg Self-esteem Scale (RSES; Rosenberg, 1965) Self-esteem total score (negatively); Suicidal Thoughts was correlated (*p*<.005) with the SIDAS (Spijker et al., 2014) total score; and Anhedonia was correlated (*p* <.05) with BFI Extraversion. These results are suggestive that the derived granular depression domain scores are sufficiently distinct from each other to warrant examining each separately, and generally exhibit acceptable construct validity given demonstrated convergence with associated constructs.

**Supplementary Materials II: Examining Expectancy**

To more rigorously test differential between-condition response on outcomes, we additionally controlled for the role of expectancy, using patients’ subjective scores of treatment-specific expectancy collected at baseline. It is plausible that such expectancy could artifactually amplify between-condition response as patients in the PT arm, who exhibit high expectancy favoring PT, may overstate response to PT, whereas patients in the escitalopram arm, who also exhibit high expectancy favoring PT, may understate response to escitalopram. Controlling for expectancy should thus enable a more rigorous evaluation of the true between-condition difference.

***The effect of expectancy on between-condition changes in Depression facets***

Results in the main text showed evidence of PT being differentially associated with reductions in *Depressed mood* and *Anhedonia*. To probe the possible confounding effect of expectancies favorable to PT, *Relative expectancy* was added as a moderator to models containing significant outcomes. No significant moderation was observed. However, statistical power was extremely low due to limited sample size, making it more likely that a true effect was not detectable. An examination of the size of the interaction term coefficients was suggestive that *Relative expectancy* had a minimal influence on the between-condition differences in *Anhedonia* (*b*_int_ = -.03, p = 909), but possibly a larger influence on the condition difference in *Depressed mood* (*b*_int_ = -.39, p = .163). Nevertheless, the unstandardized results for the *Time* by *Condition* term in the LME models estimated that at *Relative expectancy* = 0 (i.e., level at which PT expectancy does not exceed escitalopram expectancy), significant results favoring PT survived for both *Depressed mood* (B = -.10, *p* = .019) and *Anhedonia* (B = -.13, *p* < .001). That is, at a zero level of relative expectancy, significant between-condition differences of -.10 and -.13 remained for *Depressed mood* and *Anhedonia* that favored PT. These results are provided in Table S7.

***The effect of expectancy on between-condition changes in the Depression Factor***

Results in the main text showed evidence of PT being differentially associated with reductions in *Depression Factor* scores. To probe the possible effect of expectancies favorable to PT on these results, *Relative expectancy* was added as a moderator. No significant moderation was observed (*b*_int_ = -.28, *p* = .299), and the model estimated that at zero relative expectancy, a between-condition difference of -.10 units in *Depression Factor* remained, favoring PT (*p* = .030). Expectancy results are provided in Table S7.

**Supplementary Materials III: Should clinician-rated scales be privileged over self-report scales?**

One ancillary observation of our analyses was that clinician-rated scales generally exhibited larger between-condition coefficients and smaller baseline variance than self-report scales. This pattern of results is consistent with previous research, in which clinician-rated scales have consistently shown larger effect size changes than self-report scales (Greenberg et al., 1992; Edwards et al., 1984; Lambert et al., 1986; Pinquart et al., 2007; Pinquart et al., 2006), even when comparing two measures of the same assessment category (Cuijpers et al., 2010). Recognizing that strong effects can be indicative of measurement bias in addition to responsiveness to change, clinical scientists have tended to favor an inclusive approach to measurement that utilizes self-report and clinician-rated scales. Nevertheless, we believe it is important to comment on the relative strengths and weaknesses of these formats in the present study. On one hand, we observe that clinician-rated scales can be biased toward PT therapy due to capturing not only patient expectancies favorable to psilocybin, but also similar clinician expectancies. Clinician-raters also served as ‘guides’ (i.e., psychological support persons) on the Carhart-Harris et al. trial (Carhart-Harris et al., 2021), although in most cases, raters of a given patient did not serve as a ‘guide’ or therapist for that patient. In general, clinician-rated scale results were favorable to the PT condition, and uncertainty regarding the patient’s condition allocation – i.e., blinding integrity – cannot be assumed.

As such, in line with other scholars’ observations (Aday et al., 2022), it is conceivable that expectancies influenced between-condition results on clinician-rated scales and could account for larger differential effects. However, previous trials outside of the psychedelic context show similar differences in effect size between self-report and clinician ratings.

It is also important to consider strengths of clinician-rating versus self-report. First, clinician-ratings measure in vivo symptom severity for symptom domains including sadness, agitation, or slowness exhibited within the clinical interview, whereas self-report questions strictly defer to patients themselves for their self-appraisal. The former in vivo format may be more ecologically valid. Although therapeutic rapport could plausibly influence such in vivo symptoms, it bears noting that there were no differences in patient-reported therapeutic rapport between the PT and escitalopram conditions (t-value = .971, SE = 1.325, *p* = .336) – e.g., see (Murphy et al., 2022)). Clinician-ratings are also advantageous in setting a standard baseline for assessing patients’ current symptom severity that coincides with what the patient regards as “normal” or “healthy.” This may amount to an important advantage to clinician-ratings, as many self-report measures (62% of BDI_IA_ items, 67% of QIDS-SR_16_ items) defer to the patient to set a baseline for symptom severity that is considered “usual” or normal for them, and this “normal” may vary considerably between patients and over time.

In sum, both self-report and clinician-rating scales contain methodological strengths and weaknesses. In the context of psychedelic research where blinding is easily compromised and expectancy effects may play a role in moderating response (Aday et al., 2022), it may be important to innovate novel trial designs that better mask the experimental condition. In addition, mechanistic work will be especially important as this could enable stronger inferences to be drawn about the causes of clinical outcomes that cannot be sufficiently achieved by blinding alone (see (Carhart-Harris et al., 2021) for a relevant discussion).

Although clinician expectancies may have contributed to differential response on the MADRS and HRS, it nevertheless bears noting that the BDI_IA_ also showed convergent results with the MADRS and HRS, albeit at a slightly smaller effect size, and other efficacy relevant outcome measures not focused on here, also significantly favored PT.

It is encouraged that future psychedelic-assisted trials use clinician-raters who are external from patient treatment to limit expectancy effects, and that clinician expectancies, biases, and blinding are adequately measured – even if blinding integrity cannot be maintained.

**Tables**

Table S1.

Allocation of Depression Items to Ballard et al. (2018) Factors

| Factor / Item | Item Number | Status | Reason for Exclusion |  |
| --- | --- | --- | --- | --- |
| **Amotivation** |  |  |  |  |
| BDI Dissatisfaction with Life | BDI4 | Included |  |  |
| BDI Loss of Interest in People | BDI12 | Included |  |  |
| BDI Indecisiveness | BDI13 | Included |  |  |
| BDI Inability to Work | BDI15 | Included |  |  |
| BDI Irritability | BDI11 | Included |  |  |
| QIDS General interests | QIDS13 | New |  |  |
| QIDS Energy Level | QIDS14 | New |  |  |
| **Reduced Appetite** |  |  |  |  |
| BDI Reduced Appetite | BDI18 | Included |  |  |
| MADRS Reduced Appetite | MADRS5 | Included |  |  |
| QIDS Decreased appetite | QIDS6 | New |  |  |
| QIDS Decreased weight | QIDS8 | New |  |  |
| BDI Weight Loss | BDI19 | New |  |  |
| HRS Weight Loss | HRS16 | Excluded | Low variance |  |
| SHAPS Reduced Enjoyment Eating | SHAPS4 | Excluded | r.drop < .20 |  |
| **Impaired Sleep** |  |  |  |  |
| BDI Insomnia | BDI16 | Included |  |  |
| HRS Middle Insomnia | HRS5 | Included |  |  |
| HRS Early Insomnia | HRS6 | Included |  |  |
| MADRS Reduced Sleep | MADRS4 | Included |  |  |
| QIDS Sleep during the Night | QIDS2 | New |  |  |
| QIDS Waking up too Early | QIDS3 | New |  |  |
| QIDS Falling Asleep | QIDS1 | Excluded | r.drop < .20 |  |
| HRS Late Insomnia | HRS4 | Excluded | r.drop < .20 |  |
| **Suicidal Thoughts** |  |  |  |  |
| BDI Suicidal Thoughts | BDI9 | Included |  |  |
| MADRS Suicidal Thoughts | MADRS10 | Included |  |  |
| HAMD Suicide | HRS3 | Included |  |  |
| QIDS Thoughts of Death or Suicide | QIDS12 | New |  |  |
| BDI Hopelessness | BDI2 | Excluded | r.drop < .20 |  |
| **Negative Cognition** |  |  |  |  |
| BDI Increased Crying | BDI10 | Included |  |  |
| BDI Disappointment in Self | BDI7 | Included |  |  |
| BDI Feelings of Punishment | BDI6 | Included |  |  |
| BDI Guilt | BDI5 | Included |  |  |
| HRS Guilt | HRS2 | Included |  |  |
| MADRS Pessimistic Thoughts | MADRS9 | Included |  |  |
| QIDS View of myself | QIDS11 | New |  |  |
| BDI Reduced Sexual Interest | BDI21 | Excluded | r.drop < .20 |  |
| BDI Self-criticism | BDI8 | Excluded | r.drop < .20 |  |
| BDI Thoughts of Failure | BDI3 | Excluded | Suppressed internal consistency |  |
| **Tension** |  |  |  |  |
| HRS Hypochondriasis | HRS15 | Included |  |  |
| BDI Hypochondriasis | BDI20 | New |  |  |
| MADRS Inner Tension | MADRS3 | Excluded | r.drop < .20 |  |
| HRS Psychological Anxiety | HRS10 | Excluded | r.drop < .20 |  |
| BDI Fatigue | BDI17 | Excluded | r.drop < .20 |  |
| **Depressed Mood** |  |  |  |  |
| BDI Sadness | BDI1 | Included |  |  |
| HRS Psychomotor Retardation | HRS8 | Included |  |  |
| MADRS Apparent Sadness | MADRS1 | Included |  |  |
| HRS Inability to Work | HRS7 | Included |  |  |
| MADRS Inability to Feel | MADRS8 | Included |  |  |
| MADRS Concentration Difficulties | MADRS6 | Included |  |  |
| HRS Depressed Mood | HRS1 | Included |  |  |
| MADRS Reported Sadness | MADRS2 | Included |  |  |
| QIDS Feeling Sad | QIDS5 | New |  |  |
| QIDS Feeling Slowed down | QIDS15 | New |  |  |
| **Anehedonia** |  |  |  |  |
| SHAPS Reduced Enjoyment TV/Radio | SHAPS1 | Included |  |  |
| SHAPS Reduced Enjoyment Family | SHAPS2 | Included |  |  |
| SHAPS Reduced Enjoyment Hobbies | SHAPS3 | Included |  |  |
| SHAPS Reduced Enjoyment Smells | SHAPS6 | Included |  |  |
| SHAPS Reduced Enjoy Others' Happiness | SHAPS7 | Included |  |  |
| SHAPS Reduced Enjoyment Appearance | SHAPS8 | Included |  |  |
| SHAPS Reduced Enjoyment Reading | SHAPS9 | Included |  |  |
| SHAPS Reduced Enjoyment Tea/Coffee | SHAPS10 | Included |  |  |
| SHAPS Reduced Enjoyment Small Pleasures | SHAPS11 | Included |  |  |
| SHAPS Reduced Enjoyment Landscape | SHAPS12 | Included |  |  |
| SHAPS Reduced Enjoyment Helping Others | SHAPS13 | Included |  |  |
| SHAPS Reduced Enjoy Receiving Praise | SHAPS14 | Included |  |  |
| **Other Items** |  |  |  |  |
| QIDS Concentration/Decision-making | QIDS10 | Excluded | Low factor relevance |  |
| QIDS Sleeping Too Much | QIDS4 | Excluded | Low factor relevance |  |
| QIDS Increased Appetite | QIDS7 | Excluded | Low factor relevance |  |
| QIDS Increased Weight | QIDS9 | Excluded | Low factor relevance |  |
| QIDS Restlessness | QIDS16 | Excluded | Low factor relevance |  |
| BDI Worthlessness | BDI14 | Excluded | Low factor relevance |  |
| HRS Insight | HRS17 | Excluded | Low fact relevance, Low variance |  |
| HRS Agitation | HRS9 | Excluded | Excluded by Ballard et al. |  |
| HRS Somatic Anxiety | HRS11 | Excluded | Excluded by Ballard et al. |  |
| HRS Somatic Symptoms Gastrointestinal | HRS12 | Excluded | Low factor relevance |  |
| HRS Somatic Symptoms General | HRS13 | Excluded | Low factor relevance |  |
| HRS Genital Symptoms | HRS14 | Excluded | Low factor relevance |  |
| MADRS Lassitude | MADRS7 | Excluded | Excluded by Ballard et al. |  |
| SHAPS Warm Bath / Refreshing Shower | SHAPS5 | Excluded | Excluded by Ballard et al. |  |
| *Note.* BDI = Beck Depression Inventory-I (Beck et al., 1961); HRS = Hamilton Rating Scale for Depression (Hamilton, 1967); MADRS = Montgomery-Asberg Depression Rating Scale (Montgomery & Asberg, 1979); QIDS = Quick Inventory of Depressive Symptomatology (Rush et al., 2003); SHAPS = Snaith-Hamilton Pleasure Scale (Snaith et al., 1995); r.drop = R psych package alpha function statistic indicating correlation between item and total score (excluding item). | | | |  |
|  |  |  |  |  |
|  |  |  |  |  |

Table S2.

Intercorrelations among Baseline Factor Scores

| Factor | Amotivation | Reduced Appetite | Impaired Sleep | Suicidal Thoughts | Negative Cognition | Depressed Mood | Anhedonia | Depression  Factor |
| --- | --- | --- | --- | --- | --- | --- | --- | --- |
| Amotivation | n=59 | .07 | .26' | .19 | .56** | .51** | .37** | .77** |
| Reduced Appetite | .07 | n=59 | -.03 | -.01 | .09 | .06 | .08 | .15 |
| Impaired Sleep | .26' | -.03 | n=59 | .19 | .11 | .24 | .08 | .38** |
| Suicidal Thoughts | .19 | -.01 | .19 | n=59 | .09 | .33* | .20 | .28' |
| Negative Cognition | .56** | .09 | .11 | .09 | n=59 | .55** | .33* | .76** |
| Depressed Mood | .51** | .06 | .24 | .33* | .55** | n=59 | .47** | .77** |
| Anhedonia | .37** | .08 | .08 | .20 | .33* | .47** | n=59 | .43** |
| Depression Factor | .77** | .15 | .38** | .28' | .76** | .77** | .43** | n=59 |
| *Note.* Depression Factor = single factor extracted from depression items; '*p*<.05; **p*<.01; ***p*<.005. | | | | | |  |  |  |

Table S3.

Allocation of Depression Items to Ballard et al. (2018) Factors

| Factor / Item | Item Number |
| --- | --- |
| **Amotivation BDI Composite** |  |
| BDI Dissatisfaction with Life | BDI4 |
| BDI Loss of Interest in People | BDI12 |
| BDI Indecisiveness | BDI13 |
| BDI Irritability | BDI11 |
| BDI Inability to Work | BDI15 |
| **Reduced Appetite QIDS Composite** |  |
| QIDS Decreased Appetite | QIDS6 |
| QIDS Decreased Weight | QIDS8 |
| **Reduced Appetite BDI Composite** |  |
| BDI Reduced appetite | BDI18 |
| BDI Weight loss | BDI19 |
| **Impaired Sleep HRS Composite** |  |
| HRS Middle Insomnia | HAMD5 |
| HRS Early Insomnia | HAMD6 |
| **Impaired Sleep QIDS Composite** |  |
| QIDS Sleep during the Night | QIDS2 |
| QIDS Waking up too Early | QIDS3 |
| **Negative Cognition BDI Composite** |  |
| BDI Increased Crying | BDI10 |
| BDI Disappointment in Self | BDI7 |
| BDI Feelings of Punishment | BDI6 |
| BDI Guilt | BDI5 |
| **Depressed Mood HRS Composite** |  |
| HRS Psychomotor Retardation | HAMD8 |
| HRS Depressed Mood | HAMD1 |
| HRS Inability to Work | HAMD7 |
| **Depressed Mood MADRS Composite** |  |
| MADRS Apparent Sadness | MADRS1 |
| MADRS Inability to Feel | MADRS8 |
| MADRS Concentration Difficulties | MADRS6 |
| MADRS Reported Sadness | MADRS2 |
| **Depressed Mood QIDS Composite** |  |
| QIDS Feeling Sad | QIDS5 |
| QIDS Feeling Slowed Down | QIDS15 |
| *Note.* BDI = Beck Depression Inventory-I (Beck et al., 1961); HRS/HAMD = Hamilton Rating Scale for Depression (Hamilton, 1967); QIDS = Quick Inventory of Depressive Symptomatology (Rush et al., 2003). | |

Table S4.

Baseline Depression Item Factor Loadings

| Item | λ | communality | uniqueness |  |
| --- | --- | --- | --- | --- |
| BDI1 Sadness | **.69** | .48 | .52 |  |
| QIDS Depressed Mood | **.69** | .48 | .52 |  |
| BDI Amotivation | **.68** | .47 | .53 |  |
| QIDS11 View of Myself | **.67** | .45 | .55 |  |
| BDI Negative Cognition | **.59** | .34 | .66 |  |
| BDI3 Thoughts of Failure | **.56** | .32 | .68 |  |
| QIDS Impaired Sleep | **.54** | .29 | .71 |  |
| QIDS13 General interests | **.52** | .27 | .73 |  |
| MADRS Depressed Mood | **.51** | .26 | .74 |  |
| QIDS10 Concentration/Decision-making | **.51** | .26 | .74 |  |
| BDI2 Hopelessness | **.49** | .24 | .76 |  |
| MADRS9 Pessimistic Thoughts | **.48** | .23 | .77 |  |
| MADRS3 Inner Tension | **.47** | .22 | .78 |  |
| HRS Depressed Mood | **.44** | .19 | .81 |  |
| QIDS1 Falling Asleep | **.41** | .17 | .83 |  |
| QIDS12 Thoughts of Death or Suicide | .38 | .14 | .86 |  |
| MADRS10 Suicidal Thoughts | .36 | .13 | .87 |  |
| HRS13 Somatic Symptoms General | .36 | .13 | .87 |  |
| MADRS7 Lassitude | .36 | .13 | .87 |  |
| BDI16 Insomnia | .35 | .12 | .88 |  |
| BDI17 Fatigue | .34 | .12 | .88 |  |
| MADRS4 Reduced Sleep | .32 | .10 | .90 |  |
| BDI14 Worthlessness | .30 | .09 | .91 |  |
| QIDS16 Restlessness | .29 | .09 | .91 |  |
| BDI9 Suicidal Thoughts | .28 | .08 | .92 |  |
| QIDS7 Increased Appetite | .27 | .07 | .93 |  |
| HRS3 Suicide | .24 | .06 | .94 |  |
| BDI21 Reduced Sexual Interest | .24 | .06 | .94 |  |
| HRS Impared Sleep | .20 | .04 | .96 |  |
| BDI Reduced Appetite | .18 | .03 | .97 |  |
| HRS4 Late Insomnia | .18 | .03 | .97 |  |
| HRS14 Genital Symptoms | .18 | .03 | .97 |  |
| QIDS9 Increased Weight | .17 | .03 | .97 |  |
| HRS12 Somatic Symptoms Gastrointestinal | .16 | .02 | .98 |  |
| MADRS5 Reduced Appetite | .14 | .02 | .98 |  |
| HRS2 Guilt | .14 | .02 | .98 |  |
| HRS10 Psychological Anxiety | .10 | .01 | .99 |  |
| QIDS Reduced Appetite | .04 | .00 | 1.00 |  |
| HRS9 Agitation | .04 | .00 | 1.00 |  |
| BDI8 Self-criticism | .02 | .00 | 1.00 |  |
| QIDS4 Sleeping Too Much | -.12 | .02 | .98 |  |
| HRS15 Hypochondriasis | -.20 | .04 | .96 |  |
| HRS11 Somatic Anxiety | -.21 | .04 | .96 |  |
| BDI20 Hypochondriasis | -.31 | .10 | .90 |  |
| *Note.* BDI = Beck Depression Inventory-I; HRS = Hamilton Rating Scale for Depression; MADRS = Montgomery-Asberg Depression Rating Scale; QIDS = Quick Inventory of Depressive Symptomatology. BDI Amotivation contains BDI4, BDI11, BDI12, BDI13, and BDI15; BDI Reduced Appetite contains BDI18 and BDI19; QIDS Reduced Appetite contains QIDS6 and QIDS8; HAMD Impaired Sleep contains HAMD5 and HAMD6; QIDS Impaired Sleep contains QIDS2 and QIDS3; BDI Negative Cognition contains BDI10, BDI5, BDI6, and BDI7; HRS Depressed Mood contains HAMD1, HAMD7, and HAMD8; MADRS Depressed Mood contains MADRS1, MADRS2, MADRS6, and MADRS8; QIDS Depressed Mood contains QIDS5 and QIDS15. Only items loading above .40 were included in the factor score. | | | |  |
|  |  |  |  |  |
|  |  |  |  |  |
|  |  |  |  |  |
|  |  |  |  |  |

Table S5.

Convergent Validity – Correlations between baseline constructs

| Factor | BFI Neuroticism | RSES Self-esteem | SIDAS | BFI Extraversion |  |
| --- | --- | --- | --- | --- | --- |
| Amotivation | .20 | -.28' | .01 | *-.13* |  |
| Reduced Appetite | .01 | .06 | .10 | -.15 |  |
| Impaired Sleep | -.02 | -.12 | .00 | -.20 |  |
| Suicidal Thoughts | .03 | -.09 | **.56**** | -.20 |  |
| Neg Cognition | **.40**** | **-.46**** | .05 | -.09 |  |
| Depressed Mood | **.26'** | **-.42**** | .14 | -.24 |  |
| Anhedonia | .08 | -.13 | -.08 | **-.30'** |  |
| Depression Factor | .31' | -.52** | .12 | -.24 |  |
| *Note.* RSES Self-esteem = Rosenberg Self-esteem Scale total score; SIDAS = Suicidal Ideation Attributes Scale total score; BFI = Big Five Inventory; Dep Factor Score = single factor extracted from depression items. Bolded = correlation hypothesized and supported; Italicized = correlation hypothesized and not supported; 'p<.05; *p<.01; **p<.005. | | | | |  |
|  |  |  |  |  |  |
|  |  |  |  |  |  |
|  |  |  |  |  |  |

Table S6.

Examining condition differences in granular domains and depression factor

|  |  | *b* | SE (*b*) | B | SE (B) | DF | t-value | p-value |
| --- | --- | --- | --- | --- | --- | --- | --- | --- |
| Amotivation | (Intercept) | .59 | .15 | .70 | .03 | 102 | 4.01 | .000 |
|  | Group | -.01 | .21 | .00 | .04 | 102 | -.05 | .963 |
|  | Time | -.95 | .17 | -.19 | .03 | 57 | -5.56 | .000 |
|  | Group x Time | -.46 | .24 | -.09 | .05 | 57 | -1.92 | .060 |
| Reduced Appetite | (Intercept) | .25 | .19 | .33 | .02 | 112 | 1.35 | .179 |
|  | Group | -.26 | .26 | -.03 | .03 | 112 | -1.00 | .320 |
|  | Time | -.35 | .25 | -.03 | .02 | 57 | -1.42 | .162 |
|  | Group x Time | .21 | .34 | .02 | .03 | 57 | .63 | .534 |
| Impaired Sleep | (Intercept) | .31 | .18 | .64 | .03 | 85 | 1.75 | .083 |
|  | Group | .07 | .25 | .01 | .05 | 85 | .27 | .791 |
|  | Time | -.46 | .16 | -.09 | .03 | 57 | -2.87 | .006 |
|  | Group x Time | -.44 | .22 | -.08 | .04 | 57 | -1.96 | .054 |
| Suicidal Thoughts | (Intercept) | .48 | .18 | .42 | .03 | 92 | 2.70 | .008 |
|  | Group | -.40 | .25 | -.06 | .04 | 92 | -1.60 | .113 |
|  | Time | -.56 | .18 | -.08 | .03 | 57 | -3.16 | .003 |
|  | Group x Time | .02 | .25 | .00 | .04 | 57 | .09 | .930 |
| Negative Cognition | (Intercept) | .50 | .16 | .60 | .03 | 106 | 3.23 | .002 |
|  | Group | .04 | .22 | .01 | .04 | 106 | .20 | .843 |
|  | Time | -.79 | .19 | -.14 | .03 | 57 | -4.18 | .000 |
|  | Group x Time | -.52 | .26 | -.09 | .05 | 57 | -1.98 | .052 |
| Depressed Mood | (Intercept) | .60 | .14 | .59 | .02 | 112 | 4.25 | .000 |
|  | Group | .01 | .20 | .00 | .03 | 112 | .04 | .970 |
|  | Time | -.87 | .19 | -.14 | .03 | 57 | -4.63 | .000 |
|  | Group x Time | -.68 | .26 | -.11 | .04 | 57 | -2.57 | .013 |
| Anhedonia | (Intercept) | -.42 | .18 | .49 | .03 | 107 | -2.29 | .024 |
|  | Group | .67 | .26 | .10 | .04 | 107 | 2.62 | .010 |
|  | Time | .48 | .22 | .07 | .03 | 57 | 2.17 | .035 |
|  | Group x Time | -.64 | .31 | -.10 | .05 | 57 | -2.05 | .045 |
| Depression Factor | (Intercept) | .63 | .15 | .65 | .02 | 108 | 4.30 | .000 |
|  | Group | -.06 | .20 | -.01 | .03 | 108 | -.30 | .764 |
|  | Time | -.91 | .18 | -.15 | .03 | 57 | -5.08 | .000 |
|  | Group x Time | -.55 | .25 | -.09 | .04 | 57 | -2.17 | .035 |

Table S7.

Examining moderation of condition difference by relative expectancy

|  |  | *b* | SE (*b*) | B | SE (B) | DF | t-value | p-value |
| --- | --- | --- | --- | --- | --- | --- | --- | --- |
| Depressed Mood | (Intercept) | .63 | .14 | .60 | .02 | 101 | 26.87 | .000 |
|  | Time | -.83 | .19 | -.13 | .03 | 51 | -4.32 | .000 |
|  | Condition | -.09 | .20 | -.01 | .03 | 101 | -.43 | .671 |
|  | Relative Expectancy | -.06 | .14 | -.01 | .02 | 101 | -.41 | .684 |
|  | Time x Condition | -.66 | .28 | -.10 | .04 | 51 | -2.41 | .019 |
|  | Time x Relative Expectancy | .25 | .19 | .04 | .03 | 51 | 1.33 | .188 |
|  | Condition x Relative Expectancy | .10 | .20 | .01 | .03 | 101 | .47 | .639 |
|  | Time x Condition x Relative Expectancy | -.39 | .28 | -.06 | .04 | 51 | -1.42 | .163 |
| Anhedonia | (Intercept) | .47 | .16 | .62 | .02 | 82 | 25.39 | .000 |
|  | Time | -.52 | .16 | -.08 | .02 | 51 | -3.23 | .002 |
|  | Condition | .06 | .23 | .01 | .04 | 82 | .27 | .790 |
|  | Relative Expectancy | .06 | .16 | .01 | .02 | 82 | .35 | .728 |
|  | Time x Condition | -.89 | .23 | -.13 | .04 | 51 | -3.85 | .000 |
|  | Time x Relative Expectancy | .15 | .16 | .02 | .02 | 51 | .94 | .352 |
|  | Condition x Relative Expectancy | -.20 | .23 | -.03 | .04 | 82 | -.87 | .389 |
|  | Time x Condition x Relative Expectancy | -.03 | .23 | .00 | .04 | 51 | -.12 | .909 |
| Depression Factor | (Intercept) | .64 | .15 | .65 | .02 | 99 | 27.31 | .000 |
|  | Time | -.86 | .19 | -.14 | .03 | 51 | -4.61 | .000 |
|  | Condition | -.12 | .21 | -.02 | .03 | 99 | -.56 | .575 |
|  | Relative Expectancy | -.05 | .14 | -.01 | .02 | 99 | -.32 | .751 |
|  | Time x Condition | -.60 | .27 | -.10 | .04 | 51 | -2.24 | .030 |
|  | Time x Relative Expectancy | .22 | .18 | .04 | .03 | 51 | 1.21 | .234 |
|  | Condition x Relative Expectancy | .05 | .21 | .01 | .03 | 99 | .22 | .823 |
|  | Time x Condition x Relative Expectancy | -.28 | .27 | -.05 | .04 | 51 | -1.05 | .299 |

Table S8.

QIDS-SR_16_ items

Falling asleep:

I never take longer than 30 minutes to fall asleep.

I take at least 30 minutes to fall asleep, less than half the time.

I take at least 30 minutes to fall asleep, more than half the time.

I take more than 60 minutes to fall asleep, more than half the time.

Sleep during the night:

I do not wake up at night.

I have a restless, light sleep with a few brief awakenings each night.

I wake up at least once a night, but go back to sleep easily.

I awaken more than once a night and stay awake for 20 minutes or more, more than half the time.

Waking up too early:

Most of the time, I awaken no more than 30 minutes before I need to get up.

More than half the time, I awaken more than 30 minutes before I need to get up.

I almost always awaken at least one hour or so before I need to, but I go back to sleep eventually.

I awaken at least one hour before I need to, and can’t go back to sleep.

Sleeping too much:

I sleep no longer than 7–8 hours/night, without napping during the day.

I sleep no longer than 10 hours in a 24-hour period including naps.

I sleep no longer than 12 hours in a 24-hour period including naps.

I sleep longer than 12 hours in a 24-hour period including naps.

Feeling sad:

I do not feel sad.

I feel sad less than half the time.

I feel sad more than half the time.

I feel sad nearly all of the time.

Decreased appetite:

There is no change in my usual appetite.

I eat somewhat less often or lesser amounts of food than usual.

I eat much less than usual and only with personal effort.

I rarely eat within a 24-hour period, and only with extreme personal effort or when other persuade me to eat.

Increased appetite:

There is no change from my usual appetite.

I feel a need to eat more frequently than usual.

I regularly eat more often and/or greater amounts of food than usual.

I feel driven to overeat both at mealtime and between meals.

Decreased weight:

I have not had a change in my weight.

I feel as if I’ve had a slight weight loss.

I have lost 2 pounds or more.

I have lost 5 pounds or more.

Increased weight:

I have not had a change in my weight.

I feel as if I've had a slight weight gain.

I have gained 2 pounds or more.

I have gained 5 pounds or more.

Concentration / decision making:

There is no change in my usual capacity to concentrate or make decisions.

I occasionally feel indecisive or find that my attention wanders.

Most of the time, I struggle to focus my attention or to make decisions.

I cannot concentrate well enough to read or cannot make even minor decisions.

View of myself:

I see myself as equally worthwhile and deserving as other people.

I am more self-blaming than usual

I largely believe that I cause problems for others.

I think almost constantly about major and minor defects in myself.

Thoughts of death or suicide:

I do not think of suicide or death.

I feel that life is empty or wonder if it’s worth living.

I think of suicide or death several times a week for several minutes.

I think of suicide or death several times a day in some detail, or I have made specific plans for suicide or have actually tried to take my life.

General interests:

There is no change from usual in how interested I am in other people or activities.

I notice that I am less interested in people or activities.

I find I have interest in only one or two of my formerly pursued activities.

I have virtually no interest in formerly pursued activities.

Energy level:

There is no change in my usual level of energy.

I get tired more easily than usual.

I have to make a big effort to start or finish my usual daily activities (for example, shopping, homework, cooking or going to work).

I really cannot carry out most of my usual daily activities because I just don't have the energy.

Feeling slowed down:

I think, speak, and move at my usual rate of speed.

I find that my thinking is slowed down or my voice sounds dull or flat.

It takes me several seconds to respond to most questions and I'm sure my thinking is slowed.

I am often unable to respond to questions without extreme effort.

Feeling restless:

I do not feel restless.

I’m often fidgety, wringing my hands, or need to shift how I am sitting.

I have impulses to move about and am quite restless.

At times, I am unable to stay seated and need to pace around.

**References**

Ballard, E. D., Yarrington, J. S., Farmer, C. A., Lener, M. S., Kadriu, B., Lally, N., ... & Zarate Jr, C. A. (2018). Parsing the heterogeneity of depression: An exploratory factor analysis across commonly used depression rating scales. *Journal of Affective Disorders*, *231*, 51-57.

Beck, A. T., Ward, C. M., Mendelson, M., Mock, J. E., & Erbaugh, J. K. (1961). An inventory for measuring depression. *Archives of General Psychiatry, 4,* 561-571.

de Winter, J. C., Dodou, D., & Wieringa, P. A. (2009). Exploratory factor analysis with small sample sizes. *Multivariate Behavioral Research*, *44*, 147-181.

Hamilton, M. A. X. (1967). Development of a rating scale for primary depressive illness. *British Journal of Social and Clinical Psychology, 6,* 278-296.

John, O. P., Donahue, E. M., & Kentle, R. L. (1991). *The* *Big five inventory.* Technical Report, University of California, Berkeley.

Montgomery, S. A., & Åsberg, M. A. R. I. E. (1979). A new depression scale designed to be sensitive to change. *The British Journal of Psychiatry, 134,* 382-389.

Rosenberg, M. (1965). Society and the adolescent self-image. Princeton, NJ: Princeton University Press.

Rush, A. J., Trivedi, M. H., Ibrahim, H. M., Carmody, T. J., Arnow, B., Klein, D. N., ... & Keller, M. B. (2003). The 16-Item Quick Inventory of Depressive Symptomatology (QIDS), clinician rating (QIDS-C), and self-report (QIDS-SR): a psychometric evaluation in patients with chronic major depression. *Biological Psychiatry, 54*, 573-583.

Snaith, R.P., Hamilton, M., Morley, S., Humayan, A., Hargreaves, D., & Trigwell, P. (1995). A scale for the assessment of hedonic tone the Snaith-Hamilton Pleasure Scale. *British Journal of Psychiatry, 167,* 99-103.

Van Spijker, B. A., Batterham, P. J., Calear, A. L., Farrer, L., Christensen, H., Reynolds, J., & Kerkhof, A. J. (2014). The Suicidal Ideation Attributes Scale (SIDAS): Community‐based validation study of a new scale for the measurement of suicidal ideation. *Suicide and Life‐Threatening Behavior*, *44*, 408-419.
